# The biliary microbiome in symptomatic cholelithiasis: a prospective comparison across distinct bacterial niches

**DOI:** 10.1186/s12866-026-05756-4

**Published:** 2026-10-06

**Authors:** Ulrich Wirth, Ramiro Vilchez-Vargas, Franziska Fuchs, Josefine Schardey, Nadine Koch, Florian Kühn, Bernhard W Renz, Jens Werner, Christian Schulz, Joachim Andrassy

**Affiliations:** 1 Department of General, Visceral and Transplantation Surgery, LMU University Hospital, LMU Medizin, Ludwig-Maximilians-Universität München, Marchioninistr. 15, 81377 Munich, Germany; 2 Department of Medicine II, LMU University Hospital, LMU Medizin, Ludwig- Maximilians-Universität München, Munich, Germany; 3 German Center for Infection Research (DZIF), Partner Site Munich, Munich, Germany

**Keywords:** Biliary microbiome, Cholelithiasis, Cholecystitis, Choledocholithiasis, Gallstone related disease, Microbial signatures

## Abstract

**Background:**

Cholelithiasis is highly prevalent in the Western world and frequently requires surgical or endoscopic intervention. However, the composition and role of the biliary microbiome remain poorly understood. This study aimed to characterize and compare bacterial communities of the biliary niche with saliva and faeces in patients with cholelithiasis.

**Methods:**

In this prospective observational study, patients with symptomatic cholecystolithiasis, chronic or acute cholecystitis, and choledocholithiasis were included. Bacterial community composition was characterized across bile, gallstone, saliva, and faecal samples using 16 S rRNA-based sequencing. Community-level and genus-level differences were assessed according to anatomical niche and clinical diagnosis, complemented by compositional sensitivity analyses and predicted functional profiling.

**Results:**

A total of 227 samples from 81 patients were analyzed. Bacterial community composition was strongly determined by anatomical niche (PERMANOVA, R² = 0.273, *P* < 0.001), with bile and gallstone communities being most closely related but remaining significantly distinct (R² = 0.146, q < 0.001). Diagnosis-associated differences were predominantly confined to the biliary compartments and remained significant after adjustment for age, explaining 7.9% of variation in bile and 14.1% in gallstones, whereas no significant associations were observed in saliva or faeces. Compositionally aware analysis with continuous age adjustment identified robust diagnosis*-*associated signals in bile, including *Escherichia/Shigella*, *Leptotrichia*, *Serratia*, and *Pseudomonas*. Biliary communities were characterized by taxa including *Enterococcus*, *Escherichia/Shigella*, *Serratia*, and *Pseudomonas*. In bile, *Escherichia/Shigella* increased with inflammatory disease, while *Pseudomonas* decreased; compositionally aware analysis confirmed diagnosis-associated differences for *Escherichia/Shigella*, *Leptotrichia*, and *Serratia*. Predicted bacterial functions showed even stronger anatomical compartmentalization (R² = 0.507, *P* = 0.001), whereas diagnosis-associated functional differences were limited to two MetaCyc pathways in gallstones. Sensitivity analyses provided no evidence that prolonged antibiotic exposure significantly affected biliary bacterial diversity or community composition.

**Conclusion:**

This comprehensive multi-niche analysis demonstrates pronounced taxonomic and predicted functional compartmentalization of bacterial communities across saliva, bile, gallstones, and faeces in patients with cholelithiasis. Diagnosis-associated alterations were predominantly observed within biliary niches, with *Escherichia/Shigella* emerging as a consistent taxon associated with inflammatory biliary disease. These findings identify anatomical niche as a major determinant of bacterial community structure and support disease-associated alterations within the biliary microbiome.

**Trial registration:**

German Clinical Trials Register (Deutsches Register Klinische Studien) DRKS00030566 (retrospectively registered 20250818).

**Supplementary Information:**

The online version contains supplementary material available at https://doi.org/10.1186/s12866-026-05756-4.

## Background

Cholelithiasis is one of the most prevalent gastrointestinal disorders in industrialized countries, affecting up to 20% of the adult population and causing substantial hepatobiliary morbidity through complications such as acute cholecystitis, choledocholithiasis, cholangitis, and biliary pancreatitis [1–3]. Although gallstone formation has traditionally been attributed primarily to cholesterol supersaturation, altered bile composition, and impaired gallbladder motility [4–7], increasing evidence suggests that microbial factors may contribute to gallstone formation and associated inflammatory disease [4–6].

Advances in sequencing technologies have challenged the traditional concept of a sterile biliary tract and revealed complex bacterial communities within bile and gallstones [4, 6, 8]. The biliary microbiome may interact with intestinal bacterial communities through the gut–liver axis, while bacteria within the biliary tract may contribute to lithogenesis through enzymatic modification of bile ingredients, alterations in bile acid metabolism, mucosal interactions, and promotion of crystal nucleation [4–7, 9, 10]. Consistent with this concept, gallstone disease has been associated with altered bacterial diversity and enrichment of potentially pathogenic taxa, including *Escherichia*, *Salmonella*, and *Helicobacter* [4, 7].

However, the ecological organization of the biliary microbiome remains poorly understood. Most studies have investigated individual sample compartments or focused on comparisons between patients with and without gallstone disease. Consequently, it remains unclear to what extent bacterial communities in bile and gallstones represent a distinct biliary ecosystem, how they relate to extra-biliary bacterial niches, and whether biliary microbial patterns differ between clinical manifestations of gallstone disease. Addressing these questions is particularly challenging because bile represents a low-biomass bacterial environment susceptible to methodological bias and contamination [7, 8, 11].

We therefore performed a prospective multi-niche microbiome analysis of saliva, bile, gallstones, and faeces in patients with symptomatic gallstone disease. By integrating taxonomic composition, and predicted bacterial functions, we aimed to characterize the biliary bacterial niche, determine its relationship to extra-biliary bacterial communities, and assess whether biliary bacterial patterns differ between clinical manifestations of gallstone disease.

## Methods

### Study design and patient cohort

Patients undergoing elective or urgent treatment for gallstone-related disease (symptomatic cholecystolithiasis, acute cholecystitis, or choledocholithiasis) at LMU University Hospital Munich between September 2021 and January 2023 were prospectively enrolled in an observational study.

A total of 81 patients were included, yielding 227 biological samples across multiple anatomical niches, including saliva (*n* = 50), bile (*n* = 78), gallstones (*n* = 48), and faeces (*n* = 51). Sample collection was performed intraoperatively at the time of laparoscopic cholecystectomy or, in patients with choledocholithiasis, during endoscopic retrograde cholangiography (ERC). Due to clinical and procedural constraints, not all sample types were available for each patient; however, complete multi-site sampling was achieved in a subset of 31 patients. RNA preservation solution (RNAlater^®^, Invitrogen) was used and samples were stored at − 80 °C until further processing.

All surgical patients underwent standard laparoscopic cholecystectomy using the four-port technique [2, 3]. Perioperative antibiotic prophylaxis (cefuroxime and metronidazole) was administered as a single preoperative dose in elective cases, whereas patients with acute cholecystitis or cholangitis (inflammatory biliary disease) received extended antibiotic treatment according to current clinical guidelines [3].

The study was conducted in accordance with the Declaration of Helsinki and Good Clinical Practice guidelines. Ethical approval was obtained from the local ethics committee (21–0404), and the study was registered in the German Clinical Trials Register (DRKS00030566). Written informed consent was obtained from all participants.

### Clinical data collection and patient stratification

Clinical data were collected, including demographic variables, indication for intervention, body mass index (BMI), histopathological findings, and relevant medical history (gastritis, prior antibiotic exposure, proton pump inhibitor use, previous ERC, and dental status). Lifestyle factors, including diet, physical activity, alcohol consumption, and smoking, were assessed using questionnaires. In patients with inflammatory biliary disease (acute cholecystitis or choledocholithiasis), prolonged therapeutic antibiotic exposure before sampling was additionally recorded and included in the analyses (Table 1).

**Table 1:** Patient demographic and disease related data

Age (years ± SD)		Symptomatic cholecystolithiasis (A) *N* = 28	Chronic cholecystitis (B) *N* = 22	Acute cholecystitis (C) *N* = 21	Choledocho-lithiasis (D) *N* = 10	All patients *N* = 81 (%)
		50.3 ± 16.1	53.4 ± 13.0	61.6 ± 14.8	66.9 ± 18.3	56.0 ± 16.1
Age group	< 49 years	13	7	3	2	25 (30.9)
	50–61 years	8	9	8	1	26 (32.1)
	> 61 years	7	6	10	7	30 (37.0)
Gender	Female	18	15	14	4	51 (63.0)
	Male	10	7	7	6	30 (37.0)
BMI (kg/m^2^)		28.1 ± 5.5	29.2 ± 5.2	25.8 ± 4.0	27.6 ± 6.0	27.8 ± 5.2
Obesity (BMI > 30 kg/m^2^)		8	7	6	3	24 (30.0)
Lifestyle	unhealthier	5	5	3	5	18 (22.2)
	healthier	23	17	18	5	63 (77.8)
Dental disease (n; )		12	8	9	6	35 (43.2)
Previous ERC (n; )		1	2	4	-	13 (16.0)
Gastritis (n; )		8	8	5	2	23 (28.4)
PPI intake (n; )		5	2	4	3	14 (17.2)
Antibiotic intake	None	19	16	13	3	51 (63.0)
	Within 6 months	8	5	7	7	27 (33.2)
	Before 6 months	1	1	1	0	3 (3.7)
	Disease-related antibiotic exposure before sampling	0	0	5	5	10 (12.3)
	Preoperative/ preinterventional antibiotic prophylaxis	28	22	16	5	71 (87.7)

ERC: endoscopic retrograde cholangiography; BMI: body mass index; PPI: proton pump inhibitor; SD standard deviation

Histopathological evaluation of resected gallbladders was used to distinguish chronic inflammatory changes. Patients were stratified based on clinical and histopathological diagnosis into four groups: symptomatic cholecystolithiasis (A), chronic cholecystitis (B), acute cholecystitis (C), and choledocholithiasis (D).

Additional stratification variables included age, obesity (BMI ≥ 30 kg/m²), and dental disease, defined as a history of tooth extraction, periodontal disease, or caries. Lifestyle profiles were categorized into “healthier” and “less healthy” patterns based on predefined criteria derived from dietary and behavioral factors.

### Sample processing and sequencing

For bacterial community analysis of saliva, bile, and gallstones, RNA was extracted as previously described to enable the identification of transcriptionally active bacteria within these low-biomass bacterial niches [11, 12]. Briefly, extraction was performed using the RNeasy kit (Qiagen) after mechanical lysis and DNase treatment (Turbo DNase, Thermo Fisher). Complementary DNA (cDNA) was synthesized using the SuperScript III First-Strand Synthesis System (Invitrogen). Faecal samples underwent DNA extraction using established protocols, following IHMS’ (International Human Microbiome Standards) “protocol Q”. For all samples, bacterial community profiling was performed by amplification of the V1–V2 region of the 16 S rRNA gene using universal primers (27 F/338R) [12, 13]. Sequencing libraries were prepared and sequenced on an Illumina MiSeq platform (2 × 300 bp paired-end reads) [11]. Negative and positive controls were included throughout sample processing to monitor contamination and technical reliability.

### Bioinformatic analysis

Bioinformatic processing was performed using the DADA2 pipeline in R (version 4.2.2) for amplicon sequence variant (ASV) inference [14]. Negative controls were included during sample processing and subjected to the same RNA extraction and sequencing workflow as the study samples. Most negative controls yielded no or only negligible sequencing reads, whereas one control yielded a relevant number of reads with a bacterial profile overlapping with those of study samples. No automated prevalence- or frequency-based contaminant filtering algorithm was applied because the majority of negative controls yielded insufficient reads for such an analysis. ASVs were therefore not globally excluded solely based on their detection in the negative control. The RNA-based 16 S approach was chosen to preferentially characterize bacterial signatures from metabolically active/intact microorganisms and to reduce the contribution of residual or cell-free bacterial DNA, although this approach does not eliminate the possibility of contamination.

Samples with fewer than 1,000 reads were excluded, and remaining samples were rarefied to the minimum sequencing depth using phyloseq [15]. Taxonomic assignment was performed using the SILVA reference database [16]. The first 200 phylotypes and phylotypes reaching > 5% abundance in any individual sample were additionally manually annotated against the NCBI type strain database to refine taxonomic resolution. Relative abundances were calculated and downstream taxonomic analyses were performed at genus level. Community composition across anatomical niches was assessed using Bray–Curtis dissimilarity and visualized by hierarchical clustering, principal coordinates analysis (PCoA), and principal component analysis (PCA) [17].

Differences in overall bacterial community composition were assessed by PERMANOVA, with homogeneity of multivariate dispersion evaluated by PERMDISP [18]. Diagnosis-associated analyses were performed within each anatomical niche. As age differed between the clinical groups, additionally age adjusted analysis was performed. To assess potential confounding by therapeutic antibiotic exposure, an additional sensitivity analysis was performed in patients with acute cholecystitis or choledocholithiasis with possible exposure to additional antibiotic therapy due to inflammatory biliary disease. The association between antibiotic exposure and overall bacterial community composition was assessed by PERMANOVA, including an additional model adjusted for diagnostic group, and differences in multivariate dispersion were evaluated by PERMDISP. Alpha diversity was assessed using the Shannon index and observed ASV richness and compared according to antibiotic exposure using Wilcoxon rank-sum tests, with additional linear models adjusted for diagnostic group. Genus-level differences were assessed using Kruskal–Wallis tests and, where applicable, clinical data were compared by use of Mann–Whitney U test. ANCOM-BC2 was additionally applied as a compositional sensitivity analysis, excluding genera with < 10% prevalence and including age as a continuous covariate in diagnosis-associated analyses. Global and pairwise tests were performed, and pseudocount sensitivity analysis was used to assess the robustness of differential-abundance findings. Multiple testing was controlled using the Benjamini–Hochberg false-discovery rate (FDR), with q < 0.05 considered significant [19]. Heatmaps included genera with a median abundance > 0 in each diagnostic group and were generated using the R packages ggplots and RColorBrewer.

Predicted functional profiling was performed using PICRUSt2 v2.6.3 and restricted to patients with complete matched sample sets (bile, gallstone, faeces, and saliva; *n* = 31 patients, 124 samples) [20]. Predicted Kyoto Encyclopedia of Genes and Genomes Ortholog, Enzyme Commission, and MetaCyc pathway abundances were generated. Differential abundance of MetaCyc pathways was assessed using MaAsLin2 v1.26.0 [21]. For diagnosis-associated pathways, stratified PICRUSt2 output was used to determine individual ASV contributions [20]

## Results

### Cohort characteristics

Overall, 81 patients were included in this prospective observational trial (Table 1): 28 with symptomatic cholecystolithiasis (group A), 22 with chronic cholecystitis (group B), 21 with acute cholecystitis (group C), and 10 suffering from choledocholithiasis (group D). A total of 227 samples were available for bacterial community analysis, including saliva (*n* = 50), bile (*n* = 78), gallstones (*n* = 48), and faeces (*n* = 51). Complete matched sample sets from all four anatomical niches were available for 31 patients, all of them female (Supplementary Table 1).

Clinical characteristics were comparable between groups with respect to sex, obesity, lifestyle, dental disease, previous ERC, gastritis, PPI intake, or antibiotic use (all *P* > 0.05). Body mass index was comparable across groups (*P* = 0.191), whereas age differed (*P* = 0.022), with older patients in the choledocholithiasis group and younger patients in the symptomatic cholecystolithiasis group. Interestingly, the bacterial community varied by age, which is consistent with previously published findings [14]. Age was therefore considered in subsequent diagnosis-associated analyses.

### Anatomical compartmentalization of bacterial communities

A total of 7,241 distinct phylotypes were identified in 227 samples, spanning 24 phyla and 417 genera, along with 119 unclassified genera. Unsupervised analysis of the bacterial community in each sample revealed that samples predominantly grouped according to their niche (Supplementary Fig. 1).

**Fig. 1:**
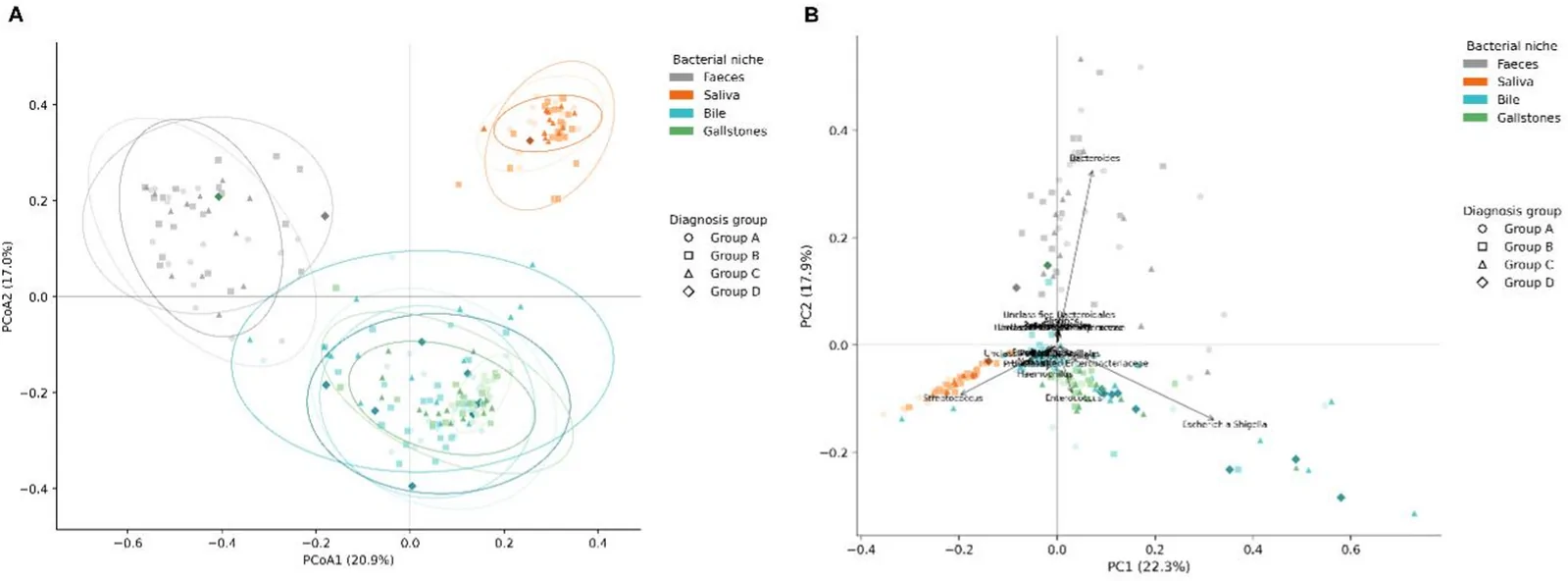
Taxonomic composition across anatomical niches. **A** Principal coordinate analysis (PCoA), **B** complemented by principal component analysis (PCA) (*n* = 81 patients) according to anatomical niche and underlying diagnosis: symptomatic cholelithiasis (A), chronic cholecystitis (B), acute cholecystitis (C), and choledocholithiasis (D)

Bray–Curtis-based PCoA confirmed a pronounced niche-specific separation of bacterial communities (Fig. 1A). Complementary PCA identified characteristic taxa contributing to this separation, including *Streptococcus* for saliva, *Bacteroides* for faeces, and *Enterococcus* for bile and gallstone communities (Fig. 1B).

**Fig. 2:**
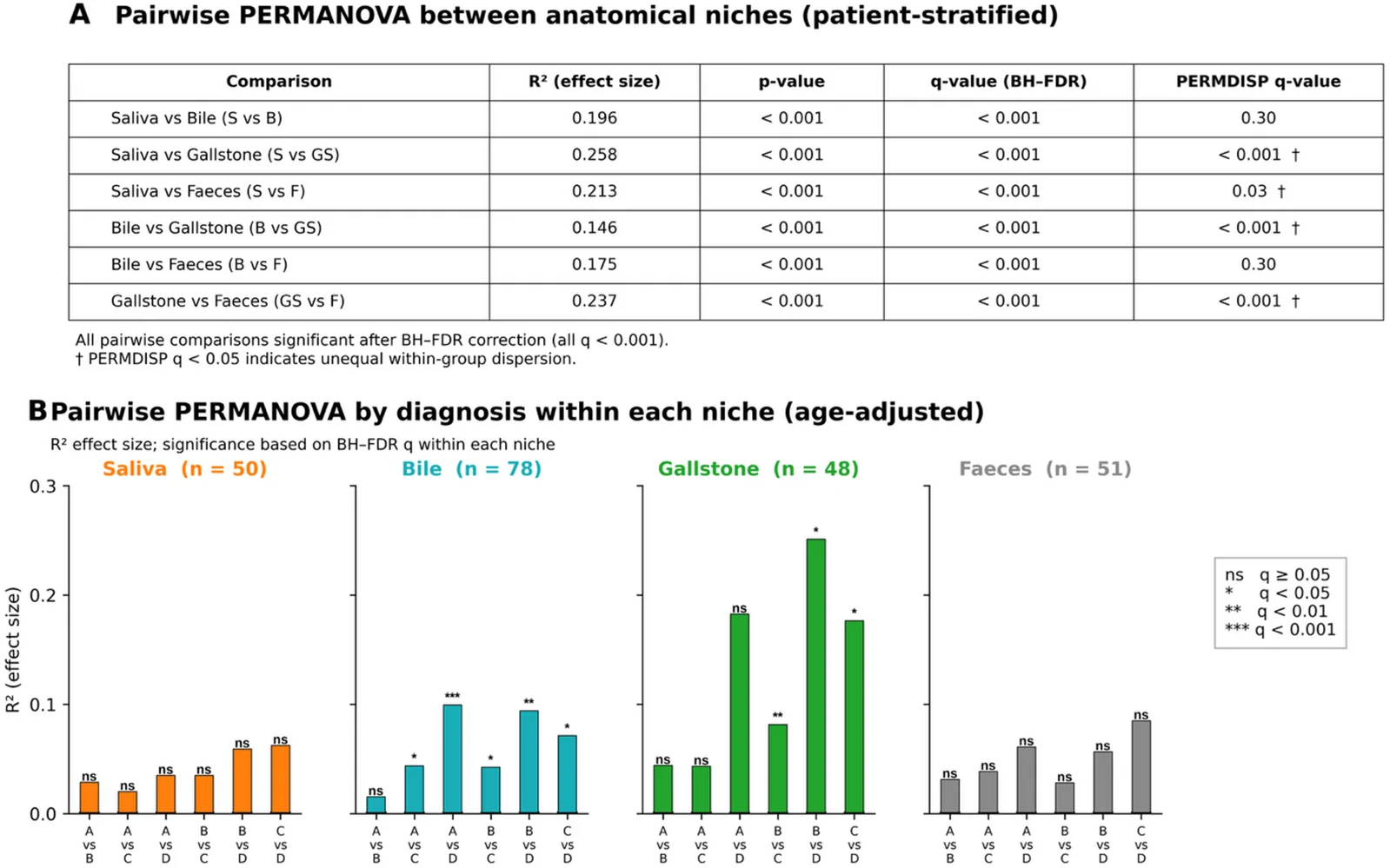
**A** pairwise PERMANOVA analysis of differences in bacterial community composition between anatomical niches in complete sample sets (*n* = 31 patients, all female) and corresponding PERMDISP results. **B** Age-adjusted PERMANOVA analysis of diagnosis-associated differences in bacterial community composition within each anatomical niche in complete sample sets (*n* = 31 patients)

Patient-stratified PERMANOVA confirmed a strong association between anatomical niche and overall bacterial composition (R² = 0.273, *P* < 0.001; Fig. 1B). All pairwise niche comparisons remained significant after FDR correction (q < 0.001), with the largest differences between saliva and gallstones (R² = 0.258) and between gallstones and faeces (R² = 0.237). Bile and gallstone communities were more closely related to each other but remained significantly distinct (R² = 0.146, *P* < 0.001, q < 0.001). PERMDISP indicated differences in within-group dispersion for several comparisons, suggesting that both community composition and heterogeneity contributed to the observed beta-diversity patterns (Fig. 2A).

Despite this pronounced compartmentalization, considerable interindividual heterogeneity was observed. Among patients with complete sample sets, a subset of bile samples clustered closer to lower gastrointestinal communities and was characterized by increased abundance of Enterobacteriaceae, particularly *Escherichia/Shigella*. Overall, approximately 15% of patients showed such lower GI-like colonization patterns, whereas most bile and gallstone samples clustered closer to upper gastrointestinal-associated communities. Samples from different niches occasionally clustered within individual patients, indicating patient-specific bacterial patterns (Supplementary Fig. 1).

Clinical subgroup analyses demonstrated that associations between diagnosis and bacterial community composition were strongly dependent on anatomical niche. After accounting for differences in age between diagnostic groups, pairwise PERMANOVA identified several diagnosis-associated differences in bile and gallstone communities after adjustment for age and FDR correction, whereas no significant pairwise differences were detected in saliva or faeces. The strongest effects were observed in gallstones, with pairwise R² values of up to approximately 0.23, followed by bile with effects of up to approximately 0.10 (Fig. 2B). Global PERMANOVA similarly identified diagnosis as the strongest clinical factor associated with bacterial composition in biliary niches, explaining 7.9% of the variation in bile (R² = 0.079, *P* < 0.001, q = 0.004) and 14.1% in gallstones (R² = 0.141, *P* < 0.001, q = 0.004), whereas no significant association was observed in saliva (R² = 0.051, *P* = 0.862) or faeces (R² = 0.066, *P* = 0.259). Age itself was not significantly associated with bacterial community composition within any anatomical compartment after adjustment for diagnosis. Other clinical variables showed weaker and predominantly non-significant associations after FDR correction (Supplementary Table 2).

To assess potential effects of antibiotic treatment on the biliary microbiome, analyses were restricted to patients with possible prolonged antibiotic exposure due to clinical diagnosis (groups C and D, Table 1). Prolonged antibiotic exposure before sample collection was not significantly associated with overall bacterial community composition (PERMANOVA, R² = 0.038, *p* = 0.239) and remained non-significant after adjustment for diagnostic group (R² = 0.031, *p* = 0.566). Multivariate dispersion did not differ significantly between antibiotic-exposed and non-exposed patients (PERMDISP, *p* = 0.156). Likewise, antibiotic exposure was not associated with Shannon diversity (*p* = 0.262) or observed ASV richness (*p* = 0.113); these associations remained non-significant after adjustment for diagnostic group (*p* = 0.729 and *p* = 0.264, respectively; Supplementary Table 3). At the genus level, none of the 148 tested taxa remained significantly associated with antibiotic exposure after correction for multiple testing (all FDR > 0.05), including Escherichia/Shigella, Enterococcus, Pseudomonas, and Serratia (Supplementary Table 4). Overall, these sensitivity analyses provided no evidence for a major effect of antibiotic exposure on biliary bacterial diversity or community composition.

### Spatial taxonomic and diagnosis-associated profiling

**Fig. 3:**
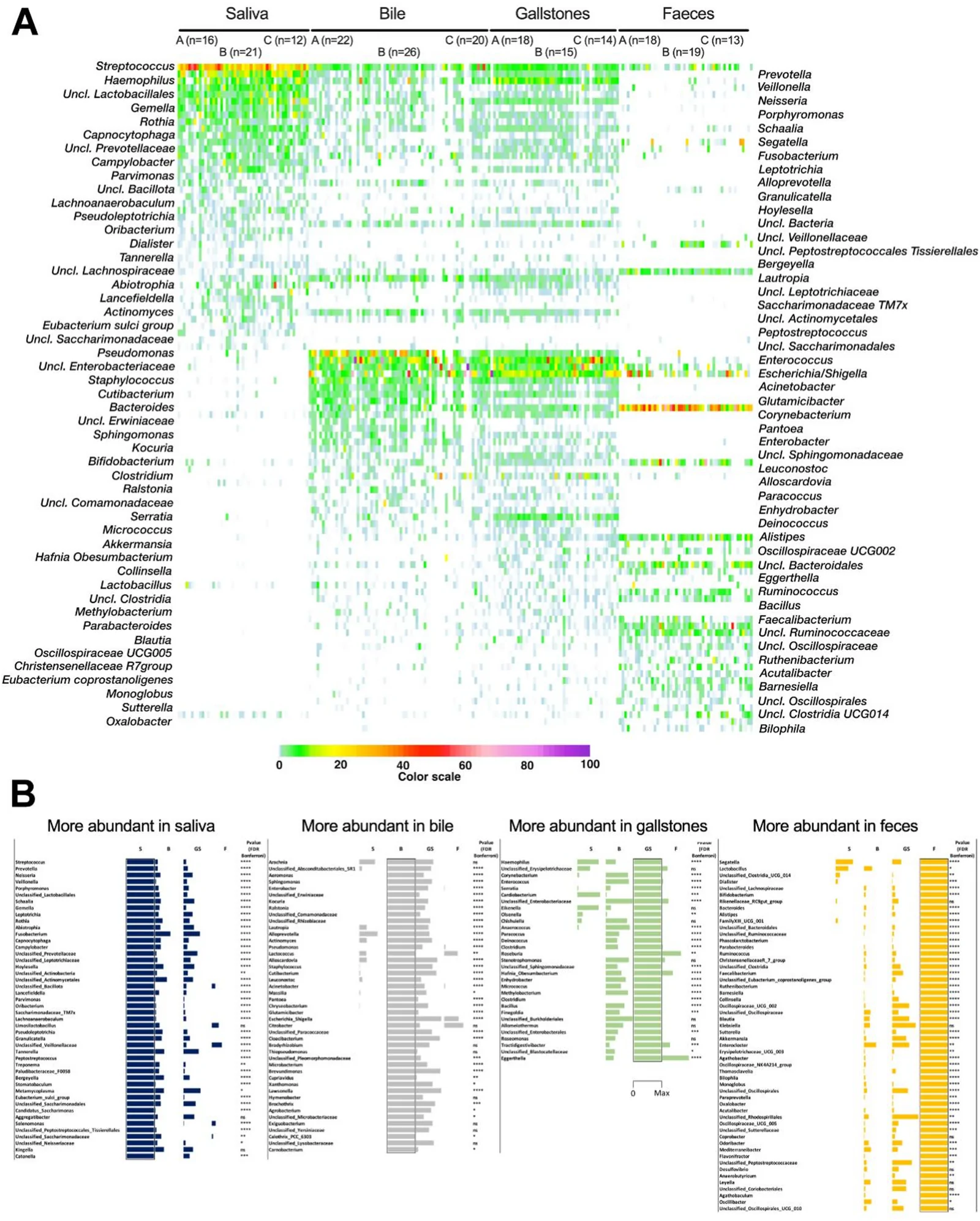
**A** heatmap of relative abundance of four different niches (saliva, bile, gallstones and faeces) stratified for underlaying disease: groups A-D: symptomatic cholecystolithiasis (A), chronic (B) or acute (C) cholecystitis and choledocholithiasis (D). **B** difference in abundance comparing the four bacterial niches

Taxonomic profiling further demonstrated distinct bacterial signatures across anatomical niches (Fig. 3A). Saliva was dominated by typical oral genera, including *Streptococcus*, *Haemophilus*, *Gemella*, *Prevotella*, *Neisseria*, and *Veillonella*, whereas faecal communities were dominated by *Bacteroides*,* Faecalibacterium*,* Bifidobacterium*, and *Segatella.* Several oral-associated genera were also detectable in biliary samples, including *Fusobacterium*, which was present in 61% of bile and 85% of gallstone samples.

Bile and gallstone communities were particularly characterized by enrichment of *Escherichia/Shigella*, *Serratia*, *Pseudomonas*, *Enterococcus*, and unclassified *Enterobacteriaceae* (Fig. 3B). *Escherichia/Shigella* showed a mean relative abundance of approximately 7% in faeces compared with 13% in bile and gallstones (*P* < 0.001) and was detected in 91% of biliary samples. *Serratia* showed a similarly pronounced biliary distribution, with a prevalence of 37% in bile and 92% in gallstones, while being nearly absent from saliva and undetectable in faeces. *Enterococcus* was detected in 92% of bile and 98% of gallstone samples. Together, these findings indicate a characteristic biliary bacterial profile that is related to, but distinct from, bacterial communities of the other gastrointestinal niches.

**Fig. 4:**
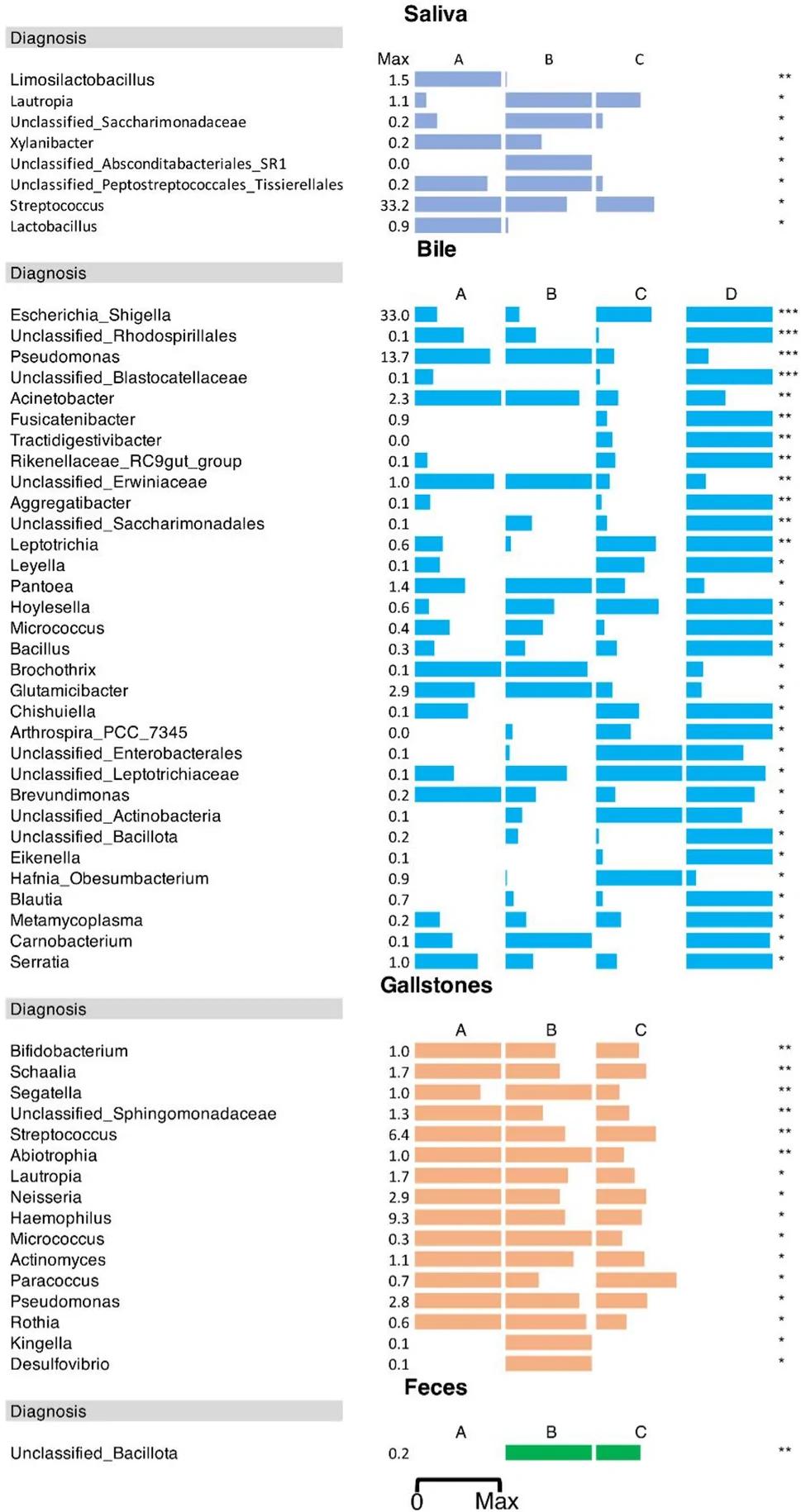
Diagnosis-specific bacterial profiles across four different niches and underlaying diagnosis groups **A-D**: symptomatic cholecystolithiasis (A), chronic (B) or acute (C) cholecystitis and choledocholithiasis (D)

Diagnosis-associated taxonomic differences were most pronounced within biliary compartments (Fig. 4, Supplementary Figs. 2–5). In bile, *Escherichia/Shigella* abundance increased with inflammatory disease and age (*P* < 0.001 and *P* < 0.01, respectively), whereas *Pseudomonas* abundance decreased, particularly in acute cholecystitis and choledocholithiasis (*P* < 0.001) (Supplementary Fig. 2). Of note, findings in the choledocholithiasis group (D) were based on bile samples obtained during ERC, whereas bile samples from the other groups were collected intraoperatively; therefore, some bias related to the sampling route cannot be excluded. Gallstone samples showed diagnosis-associated differences in several genera, including *Bifidobacterium*, *Segatella*, *Streptococcus*, *Haemophilus*, *Pseudomonas*, and *Lautropia (Supplementary Fig. **3**)*. In contrast, faecal communities showed comparatively limited diagnosis-associated variation (Supplementary Fig. 4). Saliva displayed selected differences, including higher *Streptococcus* abundance in symptomatic cholelithiasis and an opposing pattern for *Lautropia* (Supplementary Fig. 5).

To assess the robustness of diagnosis-associated taxonomic differences, compositionally aware differential abundance analysis was additionally performed using ANCOM-BC2 with age included as a covariate. Global ANCOM-BC2 analysis identified diagnosis-associated differences predominantly in bile. *Escherichia/Shigella*, *Leptotrichia*, *Serratia*, and *Pseudomonas* remained significant after FDR correction and were additionally robust in the pseudocount sensitivity analysis. No pseudocount-robust diagnosis-associated genera were identified in the other anatomical niches. (Supplementary Table 5).

### Functional profiling of bacterial communities

**Fig. 5:**
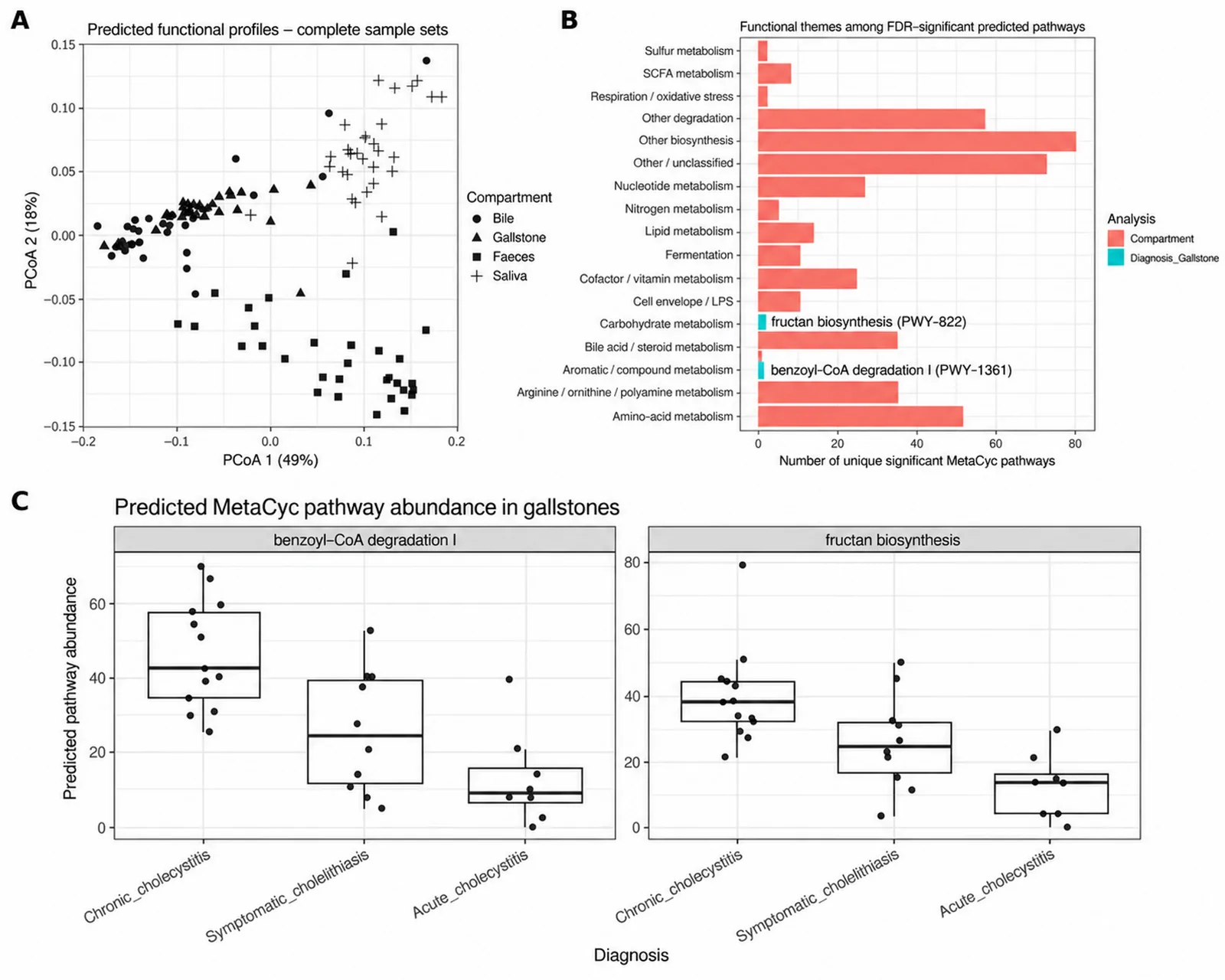
Compartment- and diagnosis-associated differences in predicted bacterial functional profiles. **A** Principal coordinate analysis (PCoA) of predicted MetaCyc pathway profiles based on complete sample sets (*n* = 31, all female). Anatomical niche was significantly associated with predicted functional composition (PERMANOVA, R² = 0.507, *P* = 0.001) **B** Functional classification of MetaCyc pathways significantly associated with anatomical compartment or diagnosis after false-discovery rate correction. **C** Predicted abundance of the two MetaCyc pathways significantly associated with diagnosis in gallstone samples, benzoyl-CoA degradation I (PWY-1361) and fructan biosynthesis (PWY-822)

Functional inference using PICRUSt2 revealed an even more pronounced compartment-specific organization of predicted bacterial functions. Anatomical niche explained 50.7% of the variation in MetaCyc pathway profiles (paired PERMANOVA, R² = 0.507, *P* = 0.001; Fig. 5A). Differential abundance analysis using MaAsLin2 identified 936 significant associations representing 462 unique MetaCyc pathways after FDR correction. These encompassed a broad range of predicted bacterial functions, including amino acid and carbohydrate metabolism, degradation pathways, nucleotide and lipid metabolism, cell-envelope-associated processes, fermentation, and nitrogen metabolism (Fig. 5B; Supplementary Fig. 6A).

In contrast to the extensive compartment-specific functional differences, associations between predicted bacterial functions and clinical diagnosis were limited. No MetaCyc pathways remained significantly associated with diagnosis after FDR correction in bile, faecal, or salivary samples. In gallstone communities, two predicted pathways differed significantly between acute and chronic cholecystitis: benzoyl-CoA degradation I (PWY-1361) and fructan biosynthesis (PWY-822), both of which were decreased in acute cholecystitis (q = 0.0206; Fig. 5C). Thus, predicted bacterial functions were predominantly determined by anatomical niche, whereas diagnosis-associated functional differences were considerably more restricted.

Stratified PICRUSt2 analysis demonstrated that both diagnosis-associated pathways were supported by multiple bacterial contributors rather than a single taxon. Although the relative contributions of individual taxa differed between diagnostic groups, none remained significantly associated with diagnosis after multiple-testing correction (Supplementary Fig. 6B). These findings suggest that the predicted functional differences reflect changes at the bacterial community rather than individual-taxon level.

## Discussion

Bacterial communities across four anatomical niches (saliva, bile, gallstones, and faeces) were analyzed in 81 patients, including 31 complete sample sets and 51 partial datasets comprising at least bile, faeces, and saliva. To our knowledge, this represents the first comprehensive comparison of these bacterial niches in patients with cholelithiasis, with a particular focus on the bacterial communities of bile and gallstones. The observed profiles revealed pronounced niche-specific patterns and provide new insights into the bacterial landscape associated with gallstone disease.

Given that bile has traditionally been considered sterile and represents a low-microbial-biomass environment, we applied an RNA-based approach following Schulz and Vasapolli to characterize transcriptionally active bacterial communities [11, 12]. In contrast to DNA-based approaches, which may additionally detect DNA derived from non-viable or degraded bacterial cells, RNA-based profiling preferentially reflects transcriptionally active bacterial populations [12, 22]. This may be particularly relevant when characterizing low-biomass bacterial communities such as those of the biliary tract [11]. The presence of a distinct and rich microbiome in stool samples is beyond question [11].

Consistent with previous studies [11], faecal samples formed a distinct cluster, while saliva samples grouped separately. Notably, bile and gallstone samples clustered closer to saliva than to faeces, suggesting greater similarity to bacterial communities of the upper gastrointestinal tract. Bile and gallstone communities were more closely related to each other than to the other anatomical niches. Although PERMANOVA indicated a significant difference between bile and gallstone communities, this difference was not confirmed by ANOSIM, suggesting that the separation between these closely related biliary niches was less robust across statistical approaches.

Clinical parameters, including PPI use, previous antibiotic exposure, and lifestyle factors, did not differ significantly between diagnostic groups and showed no consistent association with bacterial community composition. Age, however, differed significantly between diagnostic groups, with patients with choledocholithiasis representing the oldest subgroup, and therefore required particular consideration as a potential confounder. After adjustment for age, diagnosis remained significantly associated with bacterial community composition in bile and gallstone samples, indicating that these differences were not attributable to age alone. However, significant heterogeneity of dispersion among the gallstone groups indicates that the corresponding PERMANOVA result may partly reflect differences in within-group variability. Taxon-level associations were more sensitive to the statistical modelling of age. Nevertheless, compositionally aware ANCOM-BC2 analysis with age adjustment retained robust global diagnosis-associated signals in bile, including *Escherichia/Shigella*, *Leptotrichia*, *Serratia*, and *Pseudomonas*. Individual genus-level associations should therefore be interpreted cautiously.

Age itself may represent an additional determinant of biliary bacterial composition. Ageing is accompanied by changes in gastrointestinal physiology, including alterations in gastric acid secretion and bile acid metabolism as well as intestinal barrier function, which may influence bacterial composition and potentially bacterial translocation along the gut–biliary axis [23]. Thus, although diagnosis-associated differences in bile and gallstone communities remained significant after adjustment for age, residual confounding cannot be excluded, particularly given the small size and higher age of the choledocholithiasis subgroup. Importantly, conventional rank-based tests of relative abundance do not explicitly account for the compositional nature of microbiome data. We therefore complemented these analyses with ANCOM-BC2 as a compositionally aware sensitivity analysis, including age as a covariate. While several diagnosis-associated differences, particularly in bile samples, remained detectable after adjustment for age and multiple testing, other associations were not confirmed.

Previous antibiotic exposure represents another potential individual-level confounder [24]. However, it did not differ significantly between diagnostic groups and was not significantly associated with overall bacterial community composition after correction for multiple testing. Moreover, in elective cholecystectomy perioperative antibiotic prophylaxis was administered immediately before surgery, making it unlikely to account for the observed diagnosis-associated differences. In patients with possible prolonged antibiotic exposure due to inflammatory biliary disease, sensitivity analyses did not indicate a major effect of antibiotic exposure on the biliary microbiome. Antibiotic treatment showed no significant association with bacterial community composition or alpha diversity after adjustment for diagnostic group, and no individual bacterial genus was significantly associated with antibiotic exposure after multiple-testing correction. Thus, antibiotic exposure is unlikely to represent a major source of bias in the present microbiome analyses.

The taxonomic composition of the individual niches further supported the pronounced compartmentalization of the microbiome. Saliva was dominated by typical oral genera, including *Streptococcus*,* Haemophilus*,* Gemella*,* Prevotella*,* Neisseria*, and *Veillonella* [11], whereas faecal samples showed the expected predominance of intestinal taxa such as *Bacteroides*,* Faecalibacterium*,* Segatella*, and *Bifidobacterium* [11, 14]. In bile and gallstone samples, *Enterococcus*,* Serratia*,* Pseudomonas*,* Escherichia/Shigella*,* Porphyromonas*, and *Bacteroides* were frequently detected, consistent with previous reports [7, 25]. Rather than indicating direct bacterial interactions, their co-occurrence may reflect shared adaptation to the biliary environment and/or common routes of colonization. Differences between diagnostic groups were most pronounced within the biliary niches. In particular, *Escherichia/Shigella* was enriched in inflammatory disease, whereas several other taxa showed lower relative abundances. This pattern underscores the potential role of *Escherichia/Shigella* in the transition from uncomplicated cholelithiasis to inflammatory disease with displacement of taxa associated with gallstone disease [4, 6]. Importantly, not all differences identified by conventional rank-based analyses were confirmed by the compositionally aware ANCOM-BC2 analysis. The most consistent diagnosis-associated signals were observed in bile, supporting the concept that inflammatory and obstructive biliary disease is associated with alterations of the biliary bacterial community, while individual taxon-level associations should be interpreted cautiously. However, the pronounced signal involving the choledocholithiasis group must be regarded as preliminary and route-confounded, because all group D bile samples were obtained during ERC whereas the other diagnostic groups were sampled intraoperatively. Thus, an effect of endoscopic sampling, previous biliary instrumentation, or factors associated with ERC cannot be distinguished from an effect of diagnosis in this subgroup.

The similarity between bile and gallstone communities may partly reflect the selective pressures imposed by the biliary environment. Several bacteria detected in these samples, including *Enterococcus*,* Bifidobacterium*,* Lactobacillus*, and *Bacteroides*, possess bile salt hydrolases and may therefore be particularly well adapted to exposure to bile acids [26–29]. Other frequently detected taxa, including *Serratia* and members of the Enterobacteriaceae, may represent opportunistic colonizers with sufficient bile tolerance to persist within the biliary environment [30]. These findings are consistent with a bidirectional relationship between bacterial communities and bile acid metabolism, whereby bile acids shape bacterial community structure while bacterial transformation of bile acids may alter enterohepatic signaling and bile composition [4, 9].

Functional analysis complemented the taxonomic findings and demonstrated an even more pronounced compartment-specific organization of predicted bacterial functions. Anatomical niche explained approximately half of the variation in predicted MetaCyc pathway profiles, with differences spanning amino-acid and carbohydrate metabolism, degradation of aromatic compounds, nucleotide and lipid metabolism, cell-envelope-associated processes, fermentation, and nitrogen metabolism. This broad functional compartmentalization may reflect differences in local environmental conditions, including nutrient availability, bile exposure, oxygen tension, and other physicochemical properties, which are known to shape the spatial organization and functional potential of microbial communities [31, 32].

In contrast to these extensive niche-associated differences, diagnosis-associated functional alterations were limited to gallstone communities. Two predicted MetaCyc pathways, aerobic benzoyl-CoA degradation (PWY-1361) and fructan biosynthesis (PWY-822), were decreased in acute compared with chronic cholecystitis. Benzoyl-CoA degradation represents a bacterial pathway involved in the utilization of aromatic compounds, whereas fructan biosynthesis is related to bacterial carbohydrate storage and extracellular polysaccharide production. Bacterial fructans can contribute to extracellular polysaccharide production and have been implicated in nutrient storage, bacterial adhesion, and the physicochemical properties of bacterial biofilms [33]. Thus, the differential representation of this pathway may be of interest in the context of chronic gallstone-associated bacterial communities. However, its potential relevance to biliary biofilm formation or bacterial persistence remains speculative and requires functional validation [33].

Both pathways were supported by multiple bacterial contributors, with no individual taxon remaining significantly associated with diagnosis after correction for multiple testing. This suggests that the predicted functional differences may reflect community-level shifts rather than changes attributable to a single bacterial genus. Importantly, PICRUSt2 infers functional potential from 16 S-based taxonomic profiles rather than directly measuring bacterial genes, transcripts, or metabolites. Accordingly, these findings should be considered hypothesis-generating and require validation by functional multi-omics approaches.

Furthermore, several less abundant taxa warrant further investigation. *Fusobacterium* and *Porphyromonas* were abundant in saliva and also detectable in biliary samples, potentially reflecting shared bacterial sources or routes of translocation, although no association with dental status was observed in our cohort [9]. Serratia showed a particularly strong association with the biliary niches, whereas *Dialister* was detected across multiple compartments. Notably, *Dialister* has previously been reported at increased abundance in the bile of patients with cholelithiasis, and *Serratia* has also been identified as part of biliary microbial communities associated with gallstone disease [8]. These observations may reflect niche-specific selection or bacterial exchange along the gastrointestinal–biliary axis; however, the biological and clinical significance of these taxa in biliary disease remains uncertain.

This study has several limitations. Although the overall cohort size was moderate (*n* = 81), subgroup analyses were constrained by smaller sample sizes, particularly for analyses requiring complete multisite sampling (*n* = 31, all female). This selected subset may therefore not be representative of the overall cohort, as only women are included in this cohort, particularly with respect to sex, and the findings cannot necessarily be generalized to male patients. But female sex is a well-established risk factor for cholelithiasis, with gallstone prevalence being consistently higher in women than in men [3, 4]. Nevertheless, this prospective cohort provides comprehensive characterization of the biliary niche in direct comparison with oral and intestinal bacterial communities. A major limitation is the absence of biliary samples from healthy controls. But unlike saliva or faecal samples, bile cannot routinely be obtained non-invasively from healthy individuals. Consequently, inclusion of a healthy biliary control group was not feasible within the prospective study protocol. The observed biliary bacterial profiles therefore cannot be interpreted as disease-specific alterations relative to a physiological biliary microbiome, but rather as bacterial patterns and diagnosis-associated differences in patients suffering from gallstone disease.

The low bacterial biomass of bile and gallstone samples represents an additional methodological challenge, as reagent- and environment-derived sequences may disproportionately affect microbiome profiles [22]. To minimize this risk, strict sampling and processing protocols were applied and negative controls were included. Despite the inclusion of negative controls and the use of an RNA-based approach, contamination cannot be completely excluded, particularly for low-biomass biliary samples. Because most negative controls yielded insufficient sequencing reads for reliable prevalence- or frequency-based computational decontamination, residual background signals cannot be quantitatively separated from biological signals in bile and gallstone samples. Accordingly, findings from these low-biomass compartments should be interpreted with appropriate caution. A related methodological consideration is the use of different nucleic acid extraction approaches across sample types. Given the markedly different bacterial biomass and sample matrices, we employed the established RNA-based extraction protocol described by Vasapolli and Schulz for low-biomass biliary samples to focus the analysis on transcriptionally active bacterial communities, whereas DNA-based extraction was used for high-biomass faecal samples [11, 12, 22]. Although subsequent 16 S amplification, sequencing, and bioinformatic processing followed the same analytical workflow, RNA- and DNA-based approaches may capture different fractions of the bacterial community. Consequently, part of the observed differences between faecal and biliary communities may reflect methodological variation in addition to true niche-specific differences. Importantly, this methodological difference does not affect diagnosis-associated comparisons within individual niches, which constitute a major focus of the study, because all samples within each niche were processed consistently using the same methodology.

Antibiotic exposure represents a further potential confounder [24]. Neither previous nor prolonged antibiotic exposure differed significantly between diagnostic groups. Sensitivity analyses in patients with possible prolonged antibiotic treatment showed no major effect on the biliary microbiome, although individual-level effects cannot be excluded. Standardized perioperative antibiotic prophylaxis was administered shortly before intraoperative sampling and was therefore unlikely to substantially affect the observed bacterial patterns.

Finally, functional profiles were derived from 16 S-based taxonomic data using PICRUSt2 and therefore represent predicted metabolic potential rather than directly measured bacterial functions [20].

Taken together, our findings indicate that anatomical niche is the dominant determinant of taxonomic composition and predicted bacterial function, whereas diagnosis-associated differences are predominantly confined to the biliary compartments. Future studies integrating metagenomic, meta-transcriptomic, and metabolomic approaches are needed to determine whether these patterns contribute to the pathophysiology of gallstone disease or primarily reflect adaptation to the altered biliary environment.

## Conclusions

This comprehensive multi-niche analysis demonstrates pronounced taxonomic and predicted functional compartmentalization of bacterial communities across saliva, bile, gallstones, and faeces in patients with cholelithiasis. Bile and gallstone communities were most closely related to each other, while diagnosis-associated differences were predominantly observed within these biliary niches. *Escherichia/Shigella* emerged as one of the most consistent taxa associated with inflammatory biliary disease [34]. Overall, these findings identify anatomical niche as a major determinant of bacterial community structure and highlight disease-associated alterations within the biliary microbiome, providing a basis for further studies investigating their functional and pathophysiological relevance.

## Supplementary information

Below is the link to the electronic supplementary material.

Supplementary Material 1

## Data Availability

The data supporting the findings of this manuscript are available at NCBI, BioProject ID: PRJNA1499820 (http://www.ncbi.nlm.nih.gov/bioproject/1499820).

## Abbreviations

ANCOM: BC2 Analysis of Compositions of Microbiomes with Bias Correction 2
ANOSIM: Analysis of Similarities
ASV: Amplicon Sequence Variant
BMI: Body Mass Index
cDNA: Complementary DNA
DNA: Deoxyribonucleic Acid
ERC: Endoscopic Retrograde Cholangiography
FDR: False Discovery Rate
GI: Gastrointestinal
KEGG: Kyoto Encyclopedia of Genes and Genomes
LMU: Ludwig-Maximilians-Universität München
NCBI: National Center for Biotechnology Information
PCA: Principal Component Analysis
PCoA: Principal Coordinates Analysis
PERMANOVA: Permutational Multivariate Analysis of Variance
PERMDISP: Permutational Analysis of Multivariate Dispersions
PICRUSt2: Phylogenetic Investigation of Communities by Reconstruction of Unobserved States 2
PPI: Proton Pump Inhibitor
RNA: Ribonucleic Acid
rRNA: Ribosomal Ribonucleic Acid
SD: Standard Deviation
