# Supplementary material for: The biliary microbiome in symptomatic cholelithiasis: a prospective comparison across distinct bacterial niches
Source: BMC Microbiol. 2026 Oct 6;26:869. doi: 10.1186/s12866-026-05756-4 (PMC13640319; doi:10.1186/s12866-026-05756-4)
Supplement: Supplementary file 1 [file 12866_2026_5756_MOESM1_ESM.docx]

**The Biliary Microbiome in Symptomatic Cholelithiasis: A Prospective Comparison Across Microbial Niches**

*Biliary Microbiome in Cholelithiasis*

Ulrich Wirth^1Π^, Ramiro Vilchez-Vargas^2,3^, Franziska Fuchs^1^, Tianxiao Jiang^1^, Josefine Schardey^1^, Nadine Koch^2^, Helmut Arbogast^1^, Florian Kühn^1^, Bernhard W Renz^1^, Jens Werner^1^, Christian Schulz^2,3^*, Joachim Andrassy^1^*

Table of contents supplementary file:

Supplementary Table 1

Supplementary Table 2

Supplementary Table 3

Supplementary Table 4

Supplementary Table 5

Supplementary Figure 1

Supplementary Figure 2

Supplementary Figure 3

Supplementary Figure 4

Supplementary Figure 5

Supplementary Figure 6

| **Supplementary Table 1** – demographic and disease related data of patients with complete sample sets (n=31) | | | | | |
| --- | --- | --- | --- | --- | --- |
|  | | Symptomatic cholecystolithiasis (A) N=13 | Chronic cholecystitis (B) N=10 | Acute cholecystitis (C) N=8 | All patients  N=31 (%) |
| Age (years ± SD) | | 53.9 ± 14.3 | 48.9 ± 16.0 | 54.1 ± 13.1 | 52.4 ± 14.3 |
| Age group | < 49 years | 5 (38) | 4 (40) | 3 (37.5) | 12 (39) |
|  | 50-61 years | 4 (31) | 4 (40) | 3 (37.5) | 11 (35) |
|  | >61 years | 4 (31) | 2 (20) | 2 (25) | 8 (26) |
| Gender | Female | 13 (100) | 10 (100) | 8 (100) | 31 (100) |
|  | Male | 0 | 0 | 0 | 0 |
| BMI (kg/m^2^) | | 29.8 ± 6.0 | 26.8 ± 4.2 | 24.9 ± 3.8 | 27.6 ± 5.2 |
| Obesity (BMI >30kg/m^2^) | | 5 (38) | 2 (20) | 2 (25) | 9 (29) |
| Lifestyle | unhealthier | 4 (31) | 0 | 1 (12.5) | 5 (16) |
|  | healthier | 9 (69) | 10 (100) | 7 (87.5) | 26 (84) |
| Dental disease (n;) | | 5 (38) | 3 (30) | 4 (50) | 12 (39) |
| Previous ERC (n;) | | 2 (15) | 1 (10) | 2 (25) | 5 (16) |
| Gastritis (n;) | | 4 (31) | 1 (10) | 2 (25) | 7 (23) |
| PPI intake (n;) | | 2 (15) | 1 (10) | 2 (25) | 5 (16) |
| Antibiotic intake | None | 9 (69) | 8 (80) | 3 (37.5) | 22 (71) |
|  | Within 6 months | 4 (31) | 2 (20) | 5 (62.5) | 9 (29) |
|  | Before 6 months | 0 | 0 | 0 | 0 |
| ERC: endoscopic retrograde cholangiography; BMI: body mass index; PPI: proton pump inhibitor; SD standard deviation | | | | | |

**Supplementary Table 2 -** Global and pairwise comparison (PERMANOVA) between groups of the different clinical variables

| **Variable** | **Comparison** | **Oral Cavity (Saliva)** | | **Bile** | | **Gallstone** | | **Faeces** | |
| --- | --- | --- | --- | --- | --- | --- | --- | --- | --- |
|  |  | **Unadjusted** | **Age-adjusted*** | **Unadjusted** | **Age-adjusted*** | **Unadjusted** | **Age-adjusted*** | **Unadjusted** | **Age-adjusted*** |
| **Diagnosis** | **Global test** | **0.051; 0.854; 0.903** | **0.051; 0.862; 0.897†** | **0.088; <0.001; 0.004** | **0.079; <0.001; 0.005** | **0.140; <0.001; 0.004** | **0.141; <0.001; 0.005†** | **0.063; 0.369; 0.513** | **0.066; 0.259; 0.515†** |
|  | chronic cholecystitis vs. symptomatic cholelithiasis | 0.028; 0.434; 0.956 | 0.029; 0.420; 0.940 | 0.017; 0.693; 0.693 | 0.015; 0.804; 0.804 | 0.044; 0.040; 0.082 | 0.044; 0.049; 0.059 | 0.029; 0.372; 0.488 | 0.031; 0.292; 0.438 |
|  | chronic cholecystitis vs. acute cholecystitis | 0.033; 0.639; 0.956 | 0.035; 0.531; 0.940 | 0.044; 0.015; 0.018 | 0.042; 0.022; 0.026 | 0.073; 0.002; 0.010 | 0.081; <0.001; 0.004 | 0.028; 0.629; 0.629 | 0.028; 0.611; 0.611 |
|  | chronic cholecystitis vs. choledocholithiasis/ERCP | 0.057; 0.535; 0.956 | 0.059; 0.484; 0.940 | 0.111; <0.001; <0.001 | 0.094; <0.001; 0.002 | 0.250; 0.052; 0.082 | 0.251; 0.004; 0.011 | 0.064; 0.362; 0.488 | 0.056; 0.507; 0.609 |
|  | symptomatic cholelithiasis vs. acute cholecystitis | 0.021; 0.916; 0.956 | 0.020; 0.940; 0.940 | 0.046; 0.009; 0.013 | 0.044; 0.012; 0.018 | 0.043; 0.211; 0.211 | 0.043; 0.214; 0.214 | 0.033; 0.407; 0.488 | 0.038; 0.238; 0.438 |
|  | symptomatic cholelithiasis vs. choledocholithiasis/ERCP | 0.035; 0.956; 0.956 | 0.035; 0.934; 0.940 | 0.121; <0.001; <0.001 | 0.099; <0.001; <0.001 | 0.192; 0.062; 0.082 | 0.182; 0.045; 0.059 | 0.062; 0.202; 0.488 | 0.061; 0.201; 0.438 |
|  | acute cholecystitis vs. choledocholithiasis/ERCP | 0.062; 0.923; 0.956 | 0.062; 0.817; 0.940 | 0.075; 0.004; 0.009 | 0.071; 0.007; 0.014 | 0.177; 0.069; 0.082 | 0.176; 0.019; 0.039 | 0.089; 0.289; 0.488 | 0.085; 0.264; 0.438 |
| **Gender** | **Global test** | **0.028; 0.092; 0.342** | **0.028; 0.085; 0.366** | **0.021; 0.060; 0.342** | **0.021; 0.050; 0.352** | **0.026; 0.165; 0.388** | **0.023; 0.326; 0.579** | **0.021; 0.363; 0.513** | **0.021; 0.365; 0.579** |
|  | female vs. male | 0.028; 0.092; 0.092 | 0.028; 0.085; 0.085 | 0.021; 0.060; 0.060 | 0.021; 0.050; 0.050 | 0.026; 0.165; 0.165 | 0.023; 0.326; 0.326 | 0.021; 0.363; 0.363 | 0.021; 0.365; 0.365 |
| **Age groups** | **Global test** | **0.037; 0.641; 0.754** | **n.a.** | **0.030; 0.228; 0.455** | **n.a.** | **0.050; 0.159; 0.388** | **n.a.** | **0.050; 0.157; 0.388** | **n.a.** |
|  | <49 years vs. 50-61 years | 0.018; 0.953; 0.953 | n.a. | 0.020; 0.482; 0.482 | n.a. | 0.046; 0.052; 0.155 | n.a. | 0.027; 0.582; 0.582 | n.a. |
|  | <49 years vs. >61 years | 0.035; 0.254; 0.749 | n.a. | 0.022; 0.260; 0.390 | n.a. | 0.037; 0.295; 0.442 | n.a. | 0.042; 0.094; 0.194 | n.a. |
|  | 50-61 years vs. >61 years | 0.031; 0.499; 0.749 | n.a. | 0.025; 0.146; 0.390 | n.a. | 0.029; 0.662; 0.662 | n.a. | 0.045; 0.129; 0.194 | n.a. |
| **Lifestyle** | **Global test** | **0.015; 0.831; 0.903** | **0.014; 0.897; 0.897** | **0.021; 0.061; 0.342** | **0.018; 0.106; 0.366** | **0.024; 0.248; 0.473** | **0.023; 0.272; 0.544** | **0.030; 0.080; 0.342** | **0.031; 0.071; 0.364** |
|  | unhealthy vs. more healthy | 0.015; 0.831; 0.831 | 0.014; 0.897; 0.897 | 0.021; 0.061; 0.061 | 0.018; 0.106; 0.106 | 0.024; 0.248; 0.248 | 0.023; 0.272; 0.272 | 0.030; 0.080; 0.080 | 0.031; 0.071; 0.071 |
| **Alcohol consumption** | **Global test** | **0.021; 0.372; 0.513** | **0.020; 0.519; 0.682** | **0.040; 0.028; 0.279** | **0.036; 0.059; 0.352** | **0.029; 0.091; 0.342** | **0.027; 0.122; 0.366** | **0.047; 0.190; 0.422** | **0.048; 0.145; 0.401†** |
|  | no alcohol consumption vs. rare alcohol consumption | 0.021; 0.372; 0.372 | 0.020; 0.519; 0.519 | 0.021; 0.068; 0.102 | 0.019; 0.119; 0.178 | 0.029; 0.091; 0.091 | 0.027; 0.122; 0.122 | 0.029; 0.109; 0.326 | 0.030; 0.089; 0.268 |
|  | no alcohol consumption vs. regular alcohol consumption | — | — | 0.050; 0.104; 0.104 | 0.043; 0.189; 0.189 | — | — | 0.063; 0.641; 0.641 | 0.069; 0.575; 0.575 |
|  | rare alcohol consumption vs. regular alcohol consumption | — | — | 0.032; 0.062; 0.102 | 0.030; 0.076; 0.178 | — | — | 0.027; 0.501; 0.641 | 0.027; 0.474; 0.575 |
| **Dental status** | **Global test** | **0.014; 0.881; 0.903** | **0.017; 0.736; 0.897** | **0.009; 0.796; 0.903** | **0.008; 0.886; 0.897** | **0.022; 0.364; 0.513** | **0.021; 0.473; 0.682** | **0.029; 0.094; 0.342** | **0.021; 0.370; 0.579** |
|  | no dental disease vs. dental disease | 0.014; 0.881; 0.881 | 0.017; 0.736; 0.736 | 0.009; 0.796; 0.796 | 0.008; 0.886; 0.886 | 0.022; 0.364; 0.364 | 0.021; 0.473; 0.473 | 0.029; 0.094; 0.094 | 0.021; 0.370; 0.370 |
| **Gastritis** | **Global test** | **0.027; 0.124; 0.388** | **0.025; 0.189; 0.485** | **0.008; 0.920; 0.920** | **0.008; 0.895; 0.897** | **0.025; 0.208; 0.437** | **0.025; 0.215; 0.501** | **0.022; 0.316; 0.509** | **0.024; 0.223; 0.501** |
|  | no gastritis vs. gastritis | 0.027; 0.124; 0.124 | 0.025; 0.189; 0.189 | 0.008; 0.920; 0.920 | 0.008; 0.895; 0.895 | 0.025; 0.208; 0.208 | 0.025; 0.215; 0.215 | 0.022; 0.316; 0.316 | 0.024; 0.223; 0.223 |
| **PPI intake** | **Global test** | **0.020; 0.501; 0.646** | **0.022; 0.359; 0.579** | **0.015; 0.274; 0.479** | **0.012; 0.530; 0.682** | **0.027; 0.160; 0.388** | **0.033; 0.054; 0.352** | **0.020; 0.469; 0.626** | **0.012; 0.891; 0.897** |
|  | no PPI vs. PPI | 0.020; 0.501; 0.501 | 0.022; 0.359; 0.359 | 0.015; 0.274; 0.274 | 0.012; 0.530; 0.530 | 0.027; 0.160; 0.160 | 0.033; 0.054; 0.054 | 0.020; 0.469; 0.469 | 0.012; 0.891; 0.891 |
| **Antibiotics intake** | **Global test** | **0.052; 0.086; 0.342** | **0.051; 0.108; 0.366†** | **0.029; 0.275; 0.479** | **0.026; 0.406; 0.609** | **0.057; 0.126; 0.388** | **0.059; 0.112; 0.366** | **0.064; 0.014; 0.188** | **0.067; 0.008; 0.091** |
|  | no previous antibiotics vs. antibiotics within 6 months | 0.028; 0.117; 0.204 | 0.028; 0.139; 0.255 | 0.017; 0.206; 0.452 | 0.015; 0.281; 0.421 | 0.040; 0.013; 0.037 | 0.041; 0.009; 0.025 | 0.046; 0.005; 0.014 | 0.046; 0.005; 0.015 |
|  | no previous antibiotics vs. antibiotics before 6 months | 0.040; 0.136; 0.204 | 0.038; 0.181; 0.255 | 0.018; 0.477; 0.477 | 0.016; 0.618; 0.618 | 0.030; 0.277; 0.415 | 0.032; 0.231; 0.346 | 0.031; 0.396; 0.396 | 0.040; 0.153; 0.223 |
|  | antibiotics within 6 months vs. antibiotics before 6 months | 0.063; 0.272; 0.272 | 0.066; 0.255; 0.255 | 0.042; 0.301; 0.452 | 0.047; 0.213; 0.421 | 0.050; 0.522; 0.522 | 0.054; 0.443; 0.443 | 0.063; 0.324; 0.396 | 0.065; 0.223; 0.223 |
| **Obesity** | **Global test** | **0.015; 0.858; 0.903** | **0.016; 0.791; 0.897** | **0.011; 0.598; 0.747** | **0.010; 0.754; 0.897** | **0.019; 0.635; 0.754** | **0.020; 0.507; 0.682** | **0.022; 0.318; 0.509** | **0.023; 0.249; 0.528** |
|  | no obesity vs. obesity | 0.015; 0.858; 0.858 | 0.016; 0.791; 0.791 | 0.011; 0.598; 0.598 | 0.010; 0.754; 0.754 | 0.019; 0.635; 0.635 | 0.020; 0.507; 0.507 | 0.022; 0.318; 0.318 | 0.023; 0.249; 0.249 |

Values are R²; p; q (Benjamini-Hochberg FDR). * For Diagnosis, age-adjusted models use continuous age in years (Bray-Curtis ~ Age + Diagnosis; pairwise q values are corrected across all six diagnostic pairwise comparisons within each niche. Group-D pairwise diagnosis comparisons in saliva, gallstone, and faeces include one group-D sample in each niche and should therefore be interpreted cautiously. † PERMDISP q<0.05.

Abbreviations: ERCP: endoscopic retrograde cholangiopancreatography; PPI: proton pump inhibitor.

**Supplementary Table 3 -** Sensitivity analysis of potential microbiome changes associated with antibiotic exposure in biliary samples from patients in diagnostic groups C and D.

| **Analysis** | **Comparison / model** | **Effect size** | **P value** | **Interpretation / FDR** |
| --- | --- | --- | --- | --- |
| Beta diversity - PERMANOVA | Antibiotic exposure (AB vs AB_no), unadjusted | R² = 0.0382 | 0.239 | Not significant |
| Beta diversity - PERMANOVA | Antibiotic exposure, adjusted for diagnostic group | R² = 0.0313 | 0.566 | Not significant |
| Multivariate dispersion - PERMDISP | Antibiotic exposure (AB vs AB_no) | F = 2.17 | 0.156 | Not significant |
| Alpha diversity - Shannon | AB vs AB_no, Wilcoxon rank-sum test | W = 74 | 0.262 | Not significant |
| Alpha diversity - Shannon | Antibiotic exposure, adjusted for diagnostic group | β = +0.170 | 0.729 | Not significant |
| Alpha diversity - Observed ASVs | AB vs AB_no, Wilcoxon rank-sum test | W = 63.5 | 0.113 | Not significant |
| Alpha diversity - Observed ASVs | Antibiotic exposure, adjusted for diagnostic group | β = +29.48 | 0.264 | Not significant |
| Taxonomic composition | Genera significant after FDR correction (148 tested) | 0/148 | — | None significant |
| Taxonomic composition | Escherichia/Shigella | β = +0.172 | 0.209 | FDR = 0.9998 |
| Taxonomic composition | Enterococcus | β = -0.014 | 0.773 | FDR = 0.9998 |
| Taxonomic composition | Pseudomonas | β = -0.043 | 0.395 | FDR = 0.9998 |
| Taxonomic composition | Serratia | β = +0.014 | 0.523 | FDR = 0.9998 |

**Notes.** Analyses assessed differences between antibiotic-exposed (AB) and non-exposed (AB_no) patients. Beta diversity was quantified using Bray-Curtis dissimilarities. PERMANOVA and PERMDISP used 9,999 permutations. Alpha-diversity comparisons used Wilcoxon rank-sum tests. Adjusted models included diagnostic group as a covariate to account for potential confounding, but no direct comparison between diagnostic groups is reported here. Genus-level associations with antibiotic exposure were likewise adjusted for diagnostic group and corrected for multiple testing using the Benjamini-Hochberg false discovery rate.

**Supplementary Table 4 -** Genus-level associations with antibiotic exposure, adjusted for diagnostic group.

| **Genus** | **β (AB)** | **SE** | **t** | **P value** | **FDR** | **Prevalence** | **Mean relative abundance** |
| --- | --- | --- | --- | --- | --- | --- | --- |
| Sutterella | 0.0158 | 0.0044 | 3.626 | 0.0012 | 0.1747 | 13.3% | 0.018% |
| Leptotrichia | 0.0529 | 0.0207 | 2.558 | 0.0165 | 0.9998 | 43.3% | 0.505% |
| Unclassified/Oscillospirales | 0.0163 | 0.0082 | 1.988 | 0.0570 | 0.9998 | 23.3% | 0.055% |
| Rikenellaceae/RC9gut/group | 0.0127 | 0.0066 | 1.932 | 0.0639 | 0.9998 | 20.0% | 0.040% |
| Unclassified/Prevotellaceae | 0.0345 | 0.0186 | 1.853 | 0.0749 | 0.9998 | 56.7% | 0.456% |
| Corynebacterium | 0.0443 | 0.0250 | 1.770 | 0.0881 | 0.9998 | 56.7% | 0.878% |
| Unclassified/Oscillospirales/UCG/010 | -0.0071 | 0.0041 | -1.741 | 0.0931 | 0.9998 | 10.0% | 0.012% |
| Odoribacter | 0.0137 | 0.0081 | 1.699 | 0.1008 | 0.9998 | 13.3% | 0.046% |
| Akkermansia | 0.0112 | 0.0072 | 1.566 | 0.1289 | 0.9998 | 16.7% | 0.040% |
| Unclassified/Alphaproteobacteria | 0.0081 | 0.0052 | 1.565 | 0.1292 | 0.9998 | 10.0% | 0.018% |
| Ruminococcus | 0.0262 | 0.0182 | 1.438 | 0.1620 | 0.9998 | 23.3% | 0.226% |
| Prevotella | 0.0390 | 0.0273 | 1.425 | 0.1655 | 0.9998 | 66.7% | 1.177% |
| Unclassified/Bacillota | -0.0124 | 0.0089 | -1.391 | 0.1756 | 0.9998 | 13.3% | 0.061% |
| Massilia | -0.0197 | 0.0142 | -1.389 | 0.1762 | 0.9998 | 13.3% | 0.135% |
| Oribacterium | -0.0083 | 0.0061 | -1.379 | 0.1792 | 0.9998 | 10.0% | 0.024% |
| Enterobacter | -0.0277 | 0.0208 | -1.329 | 0.1948 | 0.9998 | 50.0% | 0.468% |
| Dolosigranulum | 0.0068 | 0.0052 | 1.321 | 0.1976 | 0.9998 | 13.3% | 0.021% |
| Unclassified/Mitochondria | 0.0048 | 0.0037 | 1.304 | 0.2032 | 0.9998 | 10.0% | 0.009% |
| Arthrospira/PCC/7345 | 0.0079 | 0.0060 | 1.301 | 0.2044 | 0.9998 | 16.7% | 0.028% |
| Lactococcus | -0.0205 | 0.0159 | -1.289 | 0.2084 | 0.9998 | 30.0% | 0.205% |
| Escherichia/Shigella | 0.1720 | 0.1337 | 1.286 | 0.2092 | 0.9998 | 93.3% | 25.058% |
| Faecalibacterium | -0.0078 | 0.0061 | -1.285 | 0.2097 | 0.9998 | 10.0% | 0.024% |
| Alloscardovia | 0.0171 | 0.0135 | 1.271 | 0.2145 | 0.9998 | 46.7% | 0.202% |
| Unclassified/Enterobacterales | -0.0120 | 0.0101 | -1.185 | 0.2462 | 0.9998 | 20.0% | 0.073% |
| Sphingobium | -0.0057 | 0.0048 | -1.185 | 0.2462 | 0.9998 | 10.0% | 0.015% |
| Metamycoplasma | -0.0126 | 0.0112 | -1.128 | 0.2693 | 0.9998 | 20.0% | 0.092% |
| Xanthomonas | -0.0172 | 0.0153 | -1.124 | 0.2707 | 0.9998 | 26.7% | 0.171% |
| Novosphingobium | 0.0073 | 0.0065 | 1.121 | 0.2724 | 0.9998 | 10.0% | 0.028% |
| Lachnoanaerobaculum | 0.0053 | 0.0048 | 1.104 | 0.2792 | 0.9998 | 10.0% | 0.015% |
| Unclassified/Sphingomonadaceae | 0.0228 | 0.0207 | 1.099 | 0.2813 | 0.9998 | 50.0% | 0.471% |
| Cupriavidus | -0.0141 | 0.0132 | -1.066 | 0.2958 | 0.9998 | 10.0% | 0.113% |
| Unclassified/Micrococcales | 0.0059 | 0.0056 | 1.064 | 0.2966 | 0.9998 | 10.0% | 0.021% |
| Alloprevotella | -0.0231 | 0.0217 | -1.063 | 0.2973 | 0.9998 | 53.3% | 0.508% |
| Aeromonas | -0.0490 | 0.0461 | -1.062 | 0.2978 | 0.9998 | 20.0% | 1.266% |
| Unclassified/Oscillospiraceae | -0.0145 | 0.0142 | -1.019 | 0.3173 | 0.9998 | 23.3% | 0.141% |
| Citrobacter | 0.0077 | 0.0076 | 1.006 | 0.3231 | 0.9998 | 13.3% | 0.040% |
| Unclassified/Lysobacteraceae | -0.0073 | 0.0073 | -0.999 | 0.3269 | 0.9998 | 10.0% | 0.034% |
| Oscillospiraceae/UCG/002 | 0.0036 | 0.0037 | 0.994 | 0.3291 | 0.9998 | 10.0% | 0.009% |
| Unclassified/Pleomorphomonadaceae | -0.0041 | 0.0042 | -0.976 | 0.3375 | 0.9998 | 10.0% | 0.012% |
| Aggregatibacter | 0.0054 | 0.0055 | 0.975 | 0.3381 | 0.9998 | 16.7% | 0.028% |
| Unclassified/Rhodospirillales | 0.0059 | 0.0060 | 0.972 | 0.3398 | 0.9998 | 23.3% | 0.043% |
| Collinsella | -0.0050 | 0.0052 | -0.967 | 0.3424 | 0.9998 | 10.0% | 0.018% |
| Porphyromonas | 0.0193 | 0.0201 | 0.963 | 0.3439 | 0.9998 | 56.7% | 0.480% |
| Lactobacillus | 0.0135 | 0.0146 | 0.927 | 0.3619 | 0.9998 | 40.0% | 0.174% |
| Tannerella | 0.0106 | 0.0115 | 0.920 | 0.3657 | 0.9998 | 26.7% | 0.098% |
| Pseudomonas | -0.0425 | 0.0492 | -0.863 | 0.3955 | 0.9998 | 76.7% | 3.049% |
| Enterocloster | -0.0184 | 0.0217 | -0.851 | 0.4025 | 0.9998 | 23.3% | 0.303% |
| Glutamicibacter | -0.0179 | 0.0215 | -0.830 | 0.4140 | 0.9998 | 60.0% | 0.532% |
| Unclassified/Chroococcidiopsaceae | 0.0040 | 0.0049 | 0.807 | 0.4265 | 0.9998 | 10.0% | 0.015% |
| Methylobacterium | 0.0076 | 0.0096 | 0.791 | 0.4358 | 0.9998 | 26.7% | 0.073% |
| Stenotrophomonas | 0.0064 | 0.0083 | 0.775 | 0.4449 | 0.9998 | 13.3% | 0.046% |
| Leuconostoc | -0.0121 | 0.0160 | -0.757 | 0.4557 | 0.9998 | 26.7% | 0.187% |
| Unclassified/Clostridia | 0.0110 | 0.0146 | 0.754 | 0.4571 | 0.9998 | 30.0% | 0.159% |
| Unclassified/Enterobacteriaceae | -0.0911 | 0.1220 | -0.746 | 0.4618 | 0.9998 | 86.7% | 9.807% |
| Staphylococcus | 0.0259 | 0.0354 | 0.733 | 0.4700 | 0.9998 | 80.0% | 2.373% |
| Veillonella | 0.0100 | 0.0138 | 0.722 | 0.4764 | 0.9998 | 50.0% | 0.220% |
| Limosilactobacillus | 0.0089 | 0.0124 | 0.720 | 0.4778 | 0.9998 | 20.0% | 0.104% |
| Unclassified/Rhizobiaceae | 0.0083 | 0.0115 | 0.719 | 0.4783 | 0.9998 | 43.3% | 0.128% |
| Fusobacterium | -0.0252 | 0.0352 | -0.715 | 0.4806 | 0.9998 | 70.0% | 1.245% |
| Capnocytophaga | 0.0129 | 0.0182 | 0.710 | 0.4837 | 0.9998 | 40.0% | 0.275% |
| Chishuiella | 0.0070 | 0.0098 | 0.709 | 0.4846 | 0.9998 | 23.3% | 0.073% |
| Campylobacter | -0.0116 | 0.0166 | -0.700 | 0.4900 | 0.9998 | 30.0% | 0.199% |
| Clostridium | -0.0395 | 0.0587 | -0.673 | 0.5064 | 0.9998 | 76.7% | 2.737% |
| Lachnospiraceae/NK4A136/group | 0.0036 | 0.0054 | 0.673 | 0.5067 | 0.9998 | 10.0% | 0.018% |
| Ralstonia | 0.0107 | 0.0162 | 0.660 | 0.5146 | 0.9998 | 33.3% | 0.205% |
| Desulfovibrio | 0.0049 | 0.0075 | 0.658 | 0.5160 | 0.9998 | 13.3% | 0.037% |
| Microbacterium | -0.0065 | 0.0099 | -0.656 | 0.5176 | 0.9998 | 23.3% | 0.070% |
| Serratia | 0.0140 | 0.0216 | 0.647 | 0.5231 | 0.9998 | 36.7% | 0.505% |
| Unclassified/Bacteroidales | -0.0077 | 0.0120 | -0.646 | 0.5238 | 0.9998 | 33.3% | 0.125% |
| Klebsiella | 0.0080 | 0.0125 | 0.642 | 0.5264 | 0.9998 | 16.7% | 0.104% |
| Hoylesella | 0.0141 | 0.0223 | 0.632 | 0.5329 | 0.9998 | 53.3% | 0.480% |
| Exiguobacterium | -0.0031 | 0.0050 | -0.622 | 0.5393 | 0.9998 | 13.3% | 0.018% |
| Lawsonella | 0.0058 | 0.0093 | 0.622 | 0.5395 | 0.9998 | 26.7% | 0.067% |
| Unclassified/Muribaculaceae | 0.0065 | 0.0109 | 0.593 | 0.5584 | 0.9998 | 10.0% | 0.076% |
| Brevundimonas | 0.0058 | 0.0102 | 0.574 | 0.5709 | 0.9998 | 33.3% | 0.101% |
| Unclassified/Lachnospiraceae | -0.0117 | 0.0210 | -0.560 | 0.5801 | 0.9998 | 56.7% | 0.410% |
| Leyella | 0.0045 | 0.0081 | 0.557 | 0.5823 | 0.9998 | 26.7% | 0.052% |
| Sphingobacterium | -0.0031 | 0.0057 | -0.542 | 0.5926 | 0.9998 | 13.3% | 0.021% |
| Streptococcus | 0.0427 | 0.0797 | 0.536 | 0.5963 | 0.9998 | 83.3% | 8.015% |
| Acinetobacter | 0.0126 | 0.0237 | 0.531 | 0.5998 | 0.9998 | 66.7% | 0.743% |
| Unclassified/Microbacteriaceae | -0.0068 | 0.0130 | -0.522 | 0.6060 | 0.9998 | 10.0% | 0.101% |
| ChristensenellaceaeR/7/group | 0.0034 | 0.0066 | 0.521 | 0.6067 | 0.9998 | 20.0% | 0.031% |
| Bacillus | 0.0067 | 0.0134 | 0.504 | 0.6185 | 0.9998 | 33.3% | 0.174% |
| Eubacterium/sulci/group | -0.0061 | 0.0130 | -0.468 | 0.6436 | 0.9998 | 10.0% | 0.104% |
| Abiotrophia | 0.0086 | 0.0186 | 0.465 | 0.6458 | 0.9998 | 56.7% | 0.407% |
| Lautropia | -0.0192 | 0.0416 | -0.462 | 0.6476 | 0.9998 | 80.0% | 2.498% |
| Unclassified/Pasteurellaceae | -0.0035 | 0.0078 | -0.448 | 0.6576 | 0.9998 | 13.3% | 0.040% |
| Paracoccus | 0.0072 | 0.0162 | 0.446 | 0.6591 | 0.9998 | 50.0% | 0.294% |
| Neisseria | 0.0149 | 0.0337 | 0.441 | 0.6629 | 0.9998 | 70.0% | 1.688% |
| Unclassified/Comamonadaceae | 0.0067 | 0.0156 | 0.430 | 0.6703 | 0.9998 | 40.0% | 0.211% |
| Bradyrhizobium | -0.0024 | 0.0057 | -0.425 | 0.6742 | 0.9998 | 13.3% | 0.021% |
| Alistipes | 0.0058 | 0.0138 | 0.418 | 0.6789 | 0.9998 | 40.0% | 0.171% |
| Unclassified/Erwiniaceae | -0.0069 | 0.0166 | -0.414 | 0.6823 | 0.9998 | 13.3% | 0.177% |
| Unclassified/Bacteria | -0.0094 | 0.0234 | -0.403 | 0.6902 | 0.9998 | 80.0% | 0.890% |
| Micrococcus | 0.0041 | 0.0107 | 0.384 | 0.7037 | 0.9998 | 33.3% | 0.147% |
| Bifidobacterium | 0.0079 | 0.0211 | 0.375 | 0.7104 | 0.9998 | 53.3% | 0.465% |
| Arachnia | -0.0017 | 0.0048 | -0.355 | 0.7257 | 0.9998 | 13.3% | 0.015% |
| Deinococcus | -0.0050 | 0.0147 | -0.342 | 0.7352 | 0.9998 | 40.0% | 0.193% |
| Haemophilus | 0.0142 | 0.0421 | 0.336 | 0.7394 | 0.9998 | 76.7% | 2.939% |
| Eikenella | 0.0018 | 0.0057 | 0.316 | 0.7545 | 0.9998 | 10.0% | 0.021% |
| Bacteroides | -0.0124 | 0.0400 | -0.309 | 0.7596 | 0.9998 | 76.7% | 2.168% |
| Oscillibacter | -0.0019 | 0.0065 | -0.295 | 0.7702 | 0.9998 | 13.3% | 0.028% |
| Bergeyella | -0.0024 | 0.0082 | -0.295 | 0.7703 | 0.9998 | 23.3% | 0.052% |
| Enterococcus | -0.0142 | 0.0488 | -0.292 | 0.7727 | 0.9998 | 86.7% | 5.951% |
| Unclassified/Actinobacteria | -0.0039 | 0.0133 | -0.291 | 0.7730 | 0.9998 | 16.7% | 0.113% |
| Anaerococcus | 0.0033 | 0.0117 | 0.281 | 0.7811 | 0.9998 | 40.0% | 0.128% |
| Unclassified/Leptotrichiaceae | 0.0031 | 0.0119 | 0.261 | 0.7958 | 0.9998 | 33.3% | 0.116% |
| Unclassified/Saccharimonadales | -0.0022 | 0.0085 | -0.260 | 0.7965 | 0.9998 | 16.7% | 0.058% |
| Gemella | 0.0047 | 0.0195 | 0.240 | 0.8123 | 0.9998 | 40.0% | 0.330% |
| Kineococcus | 0.0013 | 0.0057 | 0.234 | 0.8171 | 0.9998 | 10.0% | 0.021% |
| Allomeiothermus | -0.0025 | 0.0109 | -0.231 | 0.8190 | 0.9998 | 16.7% | 0.080% |
| Unclassified/Paracoccaceae | 0.0032 | 0.0140 | 0.225 | 0.8235 | 0.9998 | 43.3% | 0.174% |
| Unclassified/Clostridia/UCG/014 | 0.0015 | 0.0067 | 0.221 | 0.8269 | 0.9998 | 13.3% | 0.031% |
| Unclassified/Chloroplast | 0.0067 | 0.0321 | 0.208 | 0.8370 | 0.9998 | 76.7% | 1.508% |
| Unclassified/Blastocatellaceae | 0.0012 | 0.0060 | 0.203 | 0.8403 | 0.9998 | 16.7% | 0.031% |
| Hafnia/Obesumbacterium | -0.0060 | 0.0324 | -0.185 | 0.8549 | 0.9998 | 20.0% | 0.612% |
| Tractidigestivibacter | 0.0009 | 0.0055 | 0.172 | 0.8644 | 0.9998 | 13.3% | 0.021% |
| Peptoniphilus | 0.0012 | 0.0069 | 0.172 | 0.8651 | 0.9998 | 20.0% | 0.034% |
| Fusicatenibacter | 0.0034 | 0.0222 | 0.152 | 0.8803 | 0.9998 | 13.3% | 0.367% |
| Unclassified/Lactobacillales | -0.0026 | 0.0178 | -0.148 | 0.8831 | 0.9998 | 56.7% | 0.428% |
| Acutalibacter | -0.0008 | 0.0059 | -0.132 | 0.8962 | 0.9998 | 10.0% | 0.021% |
| Finegoldia | -0.0011 | 0.0084 | -0.130 | 0.8975 | 0.9998 | 30.0% | 0.061% |
| Schaalia | 0.0030 | 0.0231 | 0.130 | 0.8977 | 0.9998 | 70.0% | 0.835% |
| Microlunatus | -0.0008 | 0.0061 | -0.129 | 0.8985 | 0.9998 | 10.0% | 0.024% |
| Pantoea | -0.0028 | 0.0217 | -0.128 | 0.8991 | 0.9998 | 43.3% | 0.401% |
| Enhydrobacter | 0.0021 | 0.0165 | 0.126 | 0.9004 | 0.9998 | 43.3% | 0.248% |
| Cloacibacterium | -0.0015 | 0.0118 | -0.124 | 0.9019 | 0.9998 | 33.3% | 0.119% |
| Eggerthella | 0.0013 | 0.0105 | 0.119 | 0.9060 | 0.9998 | 23.3% | 0.080% |
| Parabacteroides | -0.0017 | 0.0148 | -0.118 | 0.9070 | 0.9998 | 30.0% | 0.165% |
| Unclassified/Actinomycetales | -0.0019 | 0.0200 | -0.097 | 0.9232 | 0.9998 | 56.7% | 0.413% |
| Pseudoleptotrichia | -0.0014 | 0.0147 | -0.097 | 0.9234 | 0.9998 | 23.3% | 0.150% |
| Lancefieldella | -0.0011 | 0.0117 | -0.094 | 0.9259 | 0.9998 | 16.7% | 0.092% |
| Unclassified/Absconditabacteriales/SR1 | -0.0016 | 0.0180 | -0.089 | 0.9295 | 0.9998 | 36.7% | 0.251% |
| Cutibacterium | -0.0023 | 0.0350 | -0.065 | 0.9485 | 0.9998 | 70.0% | 1.774% |
| Sphingomonas | -0.0016 | 0.0250 | -0.065 | 0.9486 | 0.9998 | 46.7% | 0.566% |
| Chryseobacterium | 0.0015 | 0.0264 | 0.056 | 0.9556 | 0.9998 | 26.7% | 0.434% |
| Roseomonas | -0.0002 | 0.0043 | -0.048 | 0.9617 | 0.9998 | 10.0% | 0.012% |
| Dialister | -0.0005 | 0.0108 | -0.047 | 0.9628 | 0.9998 | 13.3% | 0.073% |
| Agrobacterium | 0.0005 | 0.0141 | 0.033 | 0.9741 | 0.9998 | 20.0% | 0.128% |
| Paludibacteraceae/F0058 | -0.0004 | 0.0130 | -0.031 | 0.9751 | 0.9998 | 13.3% | 0.104% |
| Thiopseudomonas | -0.0003 | 0.0117 | -0.029 | 0.9774 | 0.9998 | 13.3% | 0.095% |
| Blautia | -0.0005 | 0.0183 | -0.027 | 0.9787 | 0.9998 | 13.3% | 0.254% |
| Actinomyces | 0.0006 | 0.0298 | 0.020 | 0.9842 | 0.9998 | 73.3% | 1.248% |
| Segatella | -0.0003 | 0.0157 | -0.016 | 0.9872 | 0.9998 | 50.0% | 0.248% |
| Unclassified/Ruminococcaceae | 0.0001 | 0.0109 | 0.008 | 0.9937 | 0.9998 | 40.0% | 0.107% |
| Kocuria | 0.0001 | 0.0170 | 0.006 | 0.9956 | 0.9998 | 56.7% | 0.309% |
| Granulicatella | -0.0000 | 0.0096 | -0.003 | 0.9978 | 0.9998 | 36.7% | 0.083% |
| Rothia | 0.0000 | 0.0200 | 0.000 | 0.9998 | 0.9998 | 66.7% | 0.560% |

**Notes.** β (AB) denotes the coefficient for antibiotic exposure (AB versus AB_no) from the genus-level model adjusted for diagnostic group. Relative abundance was arcsine-square-root transformed for modelling. Genera were retained when present in at least 10% of bile samples. A total of 148 genera were tested; 0 remained significant at FDR < 0.05.

**Supplementary Table 5 -** Comparison of conventional rank-based and compositionally aware differential abundance analyses

| **Niche** | **Genus** | **Rank-based significance** | **ANCOM-BC2 global q** | **Minimum pairwise q** | **Significant ANCOM-BC2 pairwise contrast(s)** | **Pseudo-count robust** | **Interpretation** |
| --- | --- | --- | --- | --- | --- | --- | --- |
| **Saliva** | Limosilactobacillus | ** |  |  |  |  | Not assessed after ANCOM-BC2 filtering |
| Saliva | Lautropia | * | 0.0650 | 1.0000 |  | No | Not confirmed by continuous-age-adjusted ANCOM-BC2 |
| Saliva | Unclassified_Saccharimonadaceae | * |  |  |  |  | Not assessed after ANCOM-BC2 filtering |
| Saliva | Xylanibacter | * |  |  |  |  | Not assessed after ANCOM-BC2 filtering |
| Saliva | Unclassified_Absconditabacteriales_SR1 | * |  |  |  |  | Not assessed after ANCOM-BC2 filtering |
| Saliva | Unclassified_Peptostreptococcales_Tissierellales | * |  |  |  |  | Not assessed after ANCOM-BC2 filtering |
| Saliva | Streptococcus | * | 0.9387 | 1.0000 |  | No | Not confirmed by continuous-age-adjusted ANCOM-BC2 |
| Saliva | Lactobacillus | * |  |  |  |  | Not assessed after ANCOM-BC2 filtering |
| **Bile** | Escherichia_Shigella | *** | 0.0010 | 0.2394 |  | Yes | Supported by continuous-age-adjusted ANCOM-BC2 global test; pseudo-count robust |
| Bile | Unclassified_Rhodospirillales | *** |  |  |  |  | Not assessed after ANCOM-BC2 filtering |
| Bile | Pseudomonas | *** | 0.0363 | 1.0000 |  | Yes | Supported by continuous-age-adjusted ANCOM-BC2 global test; pseudo-count robust |
| Bile | Unclassified_Blastocatellaceae | *** |  |  |  |  | Not assessed after ANCOM-BC2 filtering |
| Bile | Acinetobacter | ** | 0.8371 | 1.0000 |  | No | Not confirmed by continuous-age-adjusted ANCOM-BC2 |
| Bile | Fusicatenibacter | ** |  |  |  |  | Not assessed after ANCOM-BC2 filtering |
| Bile | Tractidigestivibacter | ** |  |  |  |  | Not assessed after ANCOM-BC2 filtering |
| Bile | Rikenellaceae_RC9gut_group | ** |  |  |  |  | Not assessed after ANCOM-BC2 filtering |
| Bile | Unclassified_Erwiniaceae | ** |  |  |  |  | Not assessed after ANCOM-BC2 filtering |
| Bile | Aggregatibacter | ** |  |  |  |  | Not assessed after ANCOM-BC2 filtering |
| Bile | Unclassified_Saccharimonadales | ** |  |  |  |  | Not assessed after ANCOM-BC2 filtering |
| Bile | Leptotrichia | ** | 0.0420 | 1.0000 |  | Yes | Supported by continuous-age-adjusted ANCOM-BC2 global test; pseudo-count robust |
| Bile | Leyella | * |  |  |  |  | Not assessed after ANCOM-BC2 filtering |
| Bile | Pantoea | * | 0.1113 | 1.0000 |  | No | Not confirmed by continuous-age-adjusted ANCOM-BC2 |
| Bile | Hoylesella | * |  |  |  |  | Not assessed after ANCOM-BC2 filtering |
| Bile | Micrococcus | * |  |  |  |  | Not assessed after ANCOM-BC2 filtering |
| Bile | Bacillus | * |  |  |  |  | Not assessed after ANCOM-BC2 filtering |
| Bile | Brochothrix | * |  |  |  |  | Not assessed after ANCOM-BC2 filtering |
| Bile | Glutamicibacter | * | 0.0884 | 1.0000 |  | No | Not confirmed by continuous-age-adjusted ANCOM-BC2 |
| Bile | Chishuiella | * |  |  |  |  | Not assessed after ANCOM-BC2 filtering |
| Bile | Arthrospira_PCC_7345 | * |  |  |  |  | Not assessed after ANCOM-BC2 filtering |
| Bile | Unclassified_Enterobacterales | * |  |  |  |  | Not assessed after ANCOM-BC2 filtering |
| Bile | Unclassified_Leptotrichiaceae | * |  |  |  |  | Not assessed after ANCOM-BC2 filtering |
| Bile | Brevundimonas | * |  |  |  |  | Not assessed after ANCOM-BC2 filtering |
| Bile | Unclassified_Actinobacteria | * |  |  |  |  | Not assessed after ANCOM-BC2 filtering |
| Bile | Unclassified_Bacillota | * |  |  |  |  | Not assessed after ANCOM-BC2 filtering |
| Bile | Eikenella | * |  |  |  |  | Not assessed after ANCOM-BC2 filtering |
| Bile | Hafnia_Obesumbacterium | * |  |  |  |  | Not assessed after ANCOM-BC2 filtering |
| Bile | Blautia | * |  |  |  |  | Not assessed after ANCOM-BC2 filtering |
| Bile | Metamycoplasma | * |  |  |  |  | Not assessed after ANCOM-BC2 filtering |
| Bile | Carnobacterium | * |  |  |  |  | Not assessed after ANCOM-BC2 filtering |
| Bile | Serratia | * | 0.0122 | 0.5272 |  | Yes | Supported by continuous-age-adjusted ANCOM-BC2 global test; pseudo-count robust |
| **Gallstone** | Bifidobacterium | ** | 0.7711 | 1.0000 |  | No | Not confirmed by continuous-age-adjusted ANCOM-BC2 |
| Gallstone | Schaalia | ** | 0.9985 | 1.0000 |  | No | Not confirmed by continuous-age-adjusted ANCOM-BC2 |
| Gallstone | Segatella | ** | 0.9643 | 1.0000 |  | No | Not confirmed by continuous-age-adjusted ANCOM-BC2 |
| Gallstone | Unclassified_Sphingomonadaceae | ** | 0.5923 | 1.0000 |  | No | Not confirmed by continuous-age-adjusted ANCOM-BC2 |
| Gallstone | Streptococcus | ** | 0.8261 | 1.0000 |  | No | Not confirmed by continuous-age-adjusted ANCOM-BC2 |
| Gallstone | Abiotrophia | ** | 0.9890 | 1.0000 |  | No | Not confirmed by continuous-age-adjusted ANCOM-BC2 |
| Gallstone | Lautropia | * | 0.9985 | 1.0000 |  | No | Not confirmed by continuous-age-adjusted ANCOM-BC2 |
| Gallstone | Neisseria | * | 0.9985 | 1.0000 |  | No | Not confirmed by continuous-age-adjusted ANCOM-BC2 |
| Gallstone | Haemophilus | * | 0.9985 | 1.0000 |  | No | Not confirmed by continuous-age-adjusted ANCOM-BC2 |
| Gallstone | Micrococcus | * |  |  |  |  | Not assessed after ANCOM-BC2 filtering |
| Gallstone | Actinomyces | * | 0.9985 | 1.0000 |  | No | Not confirmed by continuous-age-adjusted ANCOM-BC2 |
| Gallstone | Paracoccus | * | 0.1234 | 1.0000 |  | No | Not confirmed by continuous-age-adjusted ANCOM-BC2 |
| Gallstone | Pseudomonas | * | 0.7180 | 1.0000 |  | No | Not confirmed by continuous-age-adjusted ANCOM-BC2 |
| Gallstone | Rothia | * | 0.9985 | 1.0000 |  | No | Not confirmed by continuous-age-adjusted ANCOM-BC2 |
| Gallstone | Kingella | * |  |  |  |  | Not assessed after ANCOM-BC2 filtering |
| Gallstone | Desulfovibrio | * |  |  |  |  | Not assessed after ANCOM-BC2 filtering |
| **Faeces** | Unclassified_Bacillota | ** |  |  |  |  | Not assessed after ANCOM-BC2 filtering |

Diagnosis-associated genera identified by the original rank-based analysis were compared with ANCOM-BC2 results adjusted for age as a continuous variable (years) within each anatomical niche. The ANCOM-BC2 fixed-effects model was Age + Diagnosis_label, with Diagnosis_label specified as the grouping variable. Benjamini-Hochberg false-discovery-rate correction and pseudocount sensitivity analysis were applied. Genera not retained after ANCOM-BC2 prevalence/filtering steps are reported as not assessed rather than non-significant.

Significance stars are reproduced from the original rank-based supplementary figures. Diagnostic subgroup D was excluded from individual niche models when fewer than two samples remained after filtering.

**Supplementary Figure 1 - Unsupervised analysis of the bacterial community of all included samples of different niches (Saliva, Faeces, Bile, Gallstones)**

**Supplementary Figure 2 - disease-specific microbial signatures in bile**

**Supplementary Figure 3 - disease-specific microbial signatures in gallstones**

**Supplementary Figure 4 - disease-specific microbial signatures in feces**

**Supplementary Figure 5 - disease-specific microbial signatures in saliva**

**Supplementary Figure 6 - Compartment-associated predicted microbial functions and taxonomic contributors to diagnosis-associated pathways. A** Top compartment-associated predicted MetaCyc pathways identified by MaAsLin2. Coefficients indicate differences in predicted pathway abundance between anatomical compartments, with bile serving as the reference compartment. **B** Taxonomic contributors to the two diagnosis-associated MetaCyc pathways identified in gallstone samples, benzoyl-CoA degradation I (PWY-1361) and fructan biosynthesis (PWY-822), stratified by diagnostic group.
